# Improved TGIRT-seq methods for comprehensive transcriptome profiling with decreased adapter dimer formation and bias correction

**DOI:** 10.1038/s41598-019-44457-z

**Published:** 2019-05-28

**Authors:** Hengyi Xu, Jun Yao, Douglas C. Wu, Alan M. Lambowitz

**Affiliations:** 1 Institute for Cellular and Molecular Biology University of Texas at Austin, 78712 Austin, Texas USA; 2 Department of Molecular Biosciences, University of Texas at Austin, Austin, Texas 78712 USA

**Keywords:** RNA sequencing, Bioinformatics

## Abstract

Thermostable group II intron reverse transcriptases (TGIRTs) with high fidelity and processivity have been used for a variety of RNA sequencing (RNA-seq) applications, including comprehensive profiling of whole-cell, exosomal, and human plasma RNAs; quantitative tRNA-seq based on the ability of TGIRT enzymes to give full-length reads of tRNAs and other structured small ncRNAs; high-throughput mapping of post-transcriptional modifications; and RNA structure mapping. Here, we improved TGIRT-seq methods for comprehensive transcriptome profiling by rationally designing RNA-seq adapters that minimize adapter dimer formation. Additionally, we developed biochemical and computational methods for remediating 5′- and 3′-end biases, the latter based on a random forest regression model that provides insight into the contribution of different factors to these biases. These improvements, some of which may be applicable to other RNA-seq methods, increase the efficiency of TGIRT-seq library construction and improve coverage of very small RNAs, such as miRNAs. Our findings provide insight into the biochemical basis of 5′- and 3′-end biases in RNA-seq and suggest general approaches for remediating biases and decreasing adapter dimer formation.

## Introduction

High-throughput RNA sequencing (RNA-seq) has revolutionized biology and will become ever more powerful as new methods that address weaknesses and expand capabilities of current methods are developed^1–3^. A weakness of most current RNA-seq methods is their use of a retroviral reverse transcriptase (RT) to copy target RNAs into cDNAs for sequencing on various high-throughput DNA sequencing platforms^4^. Retroviral RTs have inherently low fidelity and processivity, and the extent to which these properties can be improved by protein engineering or *in vitro* evolution is limited by the retroviral RT structural framework^5^.

To address this weakness, we have been developing RNA-seq methods using the RTs encoded by mobile group II introns, bacterial retrotransposons that are evolutionary ancestors of introns and retroelements in eukaryotes^6–9^. Unlike retroviral RTs, which evolved to help retroviruses evade host defenses by introducing and propagating mutational variations^5^, group II intron RTs evolved to function in retrohoming, a retrotranposition mechanism that requires faithful synthesis of a full-length cDNA of a long, highly structured group II intron RNA^10^. Their beneficial properties for RNA-seq include high fidelity, processivity, and strand displacement activity, along with a proficient template-switching activity that is minimally dependent upon base pairing and enables the seamless attachment of RNA-seq adapters to target RNAs without RNA tailing or ligation^6,11^. Thermostable group II intron RTs (TGIRTs) from bacterial thermophiles combine these beneficial properties with the ability to function at high temperatures (60–65° C), which help melt out stable RNA secondary structures that can impede reverse transcription^6^. A recent crystal structure of a full-length TGIRT enzyme (GsI-IIC RT, a form of which is sold commercially as TGIRT-III; InGex) in active conformation with bound substrates revealed that group II intron RTs are closely related to RNA-dependent RNA polymerases, as expected for an ancestral RT, and identified a series of novel structural features that may contribute to their high fidelity and processivity^12^. These features include more constrained binding pockets than retroviral RTs for the templating RNA base, 3′ end of the DNA primer, and the incoming dNTP, as well as a larger fingers region that enables more extensive contact with the template-primer substrate than is possible for retroviral RTs^12^.

GsI-IIC RT (TGIRT-III) has been used for a variety of applications, including the comprehensive profiling of whole-cell, exosomal and plasma RNAs^7,8,13,14^; quantitative tRNA-seq based on the ability of the TGIRT enzyme to give full-length end-to-end reads of tRNAs with or without demethylase treatment^8,15,16^; determination of tRNA aminoacylation levels^17^; high-throughput mapping of post-transcriptional modifications by distinctive patterns of misincorporation^13,15,16,18–20^; identification of protein-bound RNAs by RIP-Seq or CLIP^18,21^; and RNA-structure mapping by DMS-MaPseq^22,23^ or SHAPE^24^. A study comparing TGIRT-seq to benchmark TruSeq v3 datasets of rRNA depleted (ribo-depleted) fragmented Universal Human Reference RNA (UHRR) with External RNA Control Consortium (ERCC) spike-ins showed that TGIRT-seq: (i) better recapitulates the relative abundance of mRNAs and ERCC spike-ins; (ii) is more strand-specific; (iii) gives more uniform 5′- to 3′-gene coverage and detects more splice junctions, particularly near the 5′ ends of genes, even from fragmented RNAs; and (iv) eliminates sequence biases due to random hexamer priming, which are inherent in TruSeq^7^. Other recent studies have shown that TGIRT-seq more accurately depicts the quantitative relationship between mRNAs and structured small ncRNAs than other tested methods^14^ and eliminates artifacts due to RT mispriming in RNA-seq reactions^25^.

**Figure 1:**
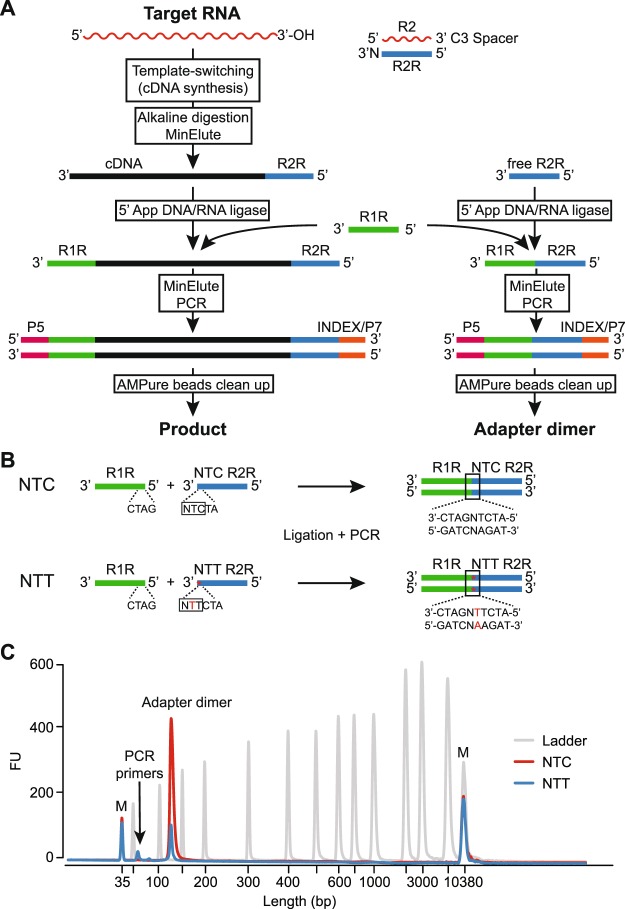
TGIRT-seq workflow and design of an improved R2R adapter that decreases adapter-dimer formation. (**A**) TGIRT-seq workflow. In the first step, TGIRT enzyme binds to an artificial template-primer substrate comprised of an RNA oligonucleotide containing an Illumina R2 sequence with a 3′-end blocking group (3SpC3) annealed to a complementary DNA oligonucleotide (R2R) that leaves a single nucleotide 3′ overhang, which can direct template-switching by base pairing to the 3′ end of an RNA template. For the preparation of TGIRT-seq libraries from pools of RNAs, the DNA primer consists of a mixture of DNA oligonucleotides that leave A, C, G, and T 3′ overhangs (denoted N). After pre-incubation of the TGIRT enzyme with the target RNAs and template-primer (see Methods), template-switching and reverse transcription of an RNA template are initiated by adding dNTPs. The resulting cDNA with an R2R adapter attached to its 5′ end is incubated with NaOH to degrade the RNA template and neutralized with HCl, followed by two rounds of MinElute clean-up using the same MinElute column (Qiagen). A pre-adenylated oligonucleotide containing the reverse complement of an Illumina R1 sequence (R1R) is then ligated to the 3′ end of the cDNA by using thermostable 5′ App DNA/RNA ligase (New England Biolabs), followed by MinElute clean-up and 12 cycles of PCR amplification with primers that add indices and capture sites for Illumina sequencing. Unused R2R adapters that are carried over from previous steps are also ligated to the R1R adapter by the 5′ App DNA/RNA ligase (New England Biolabs), resulting in the formation of adapter dimers (pathway at right), which are removed by AMPure beads clean-up prior to sequencing. (**B**) Taking into account known biases of the 5′ App DNA/RNA ligase^7,28,29^, the R2R adapter used previously in TGIRT-seq (denoted NTC) was modified by inserting a single T-residue at position −3, creating a modified R2R adapter (denoted NTT), which decreases adapter-dimer formation. (**C**) Bioanalyzer traces comparing adapter-dimer formation using the previous NTC and improved NTT R2R adapters. 2 pmole of the NTC or NTC R2R adapter was ligated to 40 pmole of adenylated R1R adapter followed by 12 cycles of PCR according to the TGIRT-seq protocol and 1 round of clean-up with 1.4X AMPure beads to remove salt, PCR primers, and adapter dimers. The products were analyzed by using a 2100 Bioanalyzer (Agilent) with a high sensitivity DNA chip. M: internal markers in the NTC (red) or NTT (blue) traces.

The TGIRT-seq method currently used for comprehensive transcriptome profiling (also referred to as TGIRT Total RNA-seq method) is outlined in Fig. 1. This method uses the ability of TGIRT enzymes to template-switch directly from an artificial RNA template/DNA primer substrate containing an RNA-seq adapter sequence to the 3′ end of an RNA template, thereby coupling RNA-seq adapter addition to the initiation of cDNA synthesis^6^. For Illumina RNA-seq, the initial RNA template/DNA primer consists of a 34-nt RNA oligonucleotide containing an Illumina Read 2 sequence (R2 RNA) with a 3′ blocking group (C3 Spacer, 3SpC3) annealed to a 35-nt DNA primer (R2R DNA) that leaves a single nucleotide 3′-DNA overhang. The latter can base pair to the 3′ end of the target RNA, serving as a springboard for TGIRT template-switching and the initiation of cDNA synthesis^6^. To capture heterogeneous 3′ ends in a pool of RNAs, this single nucleotide 3′ overhang is an equimolar mixture of A, C, G, and T (denoted N) and is added in excess to the target RNA. After reverse transcription, a second RNA-seq adapter (R1R DNA; containing the reverse complement of an Illumina Read 1 sequence) is ligated to the opposite end of the cDNA by a single-stranded DNA ligation with thermostable 5′ App RNA/DNA ligase (New England Biolabs), and this is followed by minimal PCR amplification with primers that add Illumina capture sites and sequencing indices. By avoiding gel-purification steps, TGIRT-seq libraries can be generated rapidly from small amounts of starting material (1–2 ng input RNA).

Unlike retroviral RTs, which have been studied extensively and optimized for biotechnological applications for decades, the recently introduced TGIRT enzymes and TGIRT-seq methods are potentially subject to substantial improvement. In this regard, a weakness of the TGIRT Total RNA-seq method is the thermostable 5′ App RNA/DNA ligase used to attach the R1R adapter to the 3′ end of the cDNA, which introduces sampling biases for cDNA ends and produces unwanted adapter dimers by ligating the R1R adapter to residual R2R adapter carried over from previous steps. To avoid wasting reads, these adapter dimers are removed by AMPure beads clean-up of the library prior to sequencing (Fig. 1A), a step that can result in the differential loss of sequences corresponding to miRNAs and other very small RNAs, whose library products are close in size to adapter dimers (146 and 124 nt, respectively)^14^. This problem is particularly acute for low abundance RNA samples where multiple rounds of AMPure beads clean-up may be required to sufficiently decrease the ratio of adapter dimers to a small amount of library products^8^. Due in part to this limitation, miRNAs and other very small RNAs have been analyzed by an alternative TGIRT-seq method (the TGIRT CircLigase method), which was patterned after the method used for ribosome profiling with retroviral RTs^26,27^. In the TGIRT-based version of this method, template-switching rather than RNA ligation is used to add an adapter containing both R1R and R2R sequences, and the resulting cDNAs with the linked R1R/R2R adapter are gel-purified and circularized with CircLigase for RNA-seq library construction^6,18^.

Here, we used the previously determined ligation biases of the thermostable 5′ App RNA/DNA ligase^7,28,29^ to design an R2/R2R adapter with just a single nucleotide change that dramatically decreases the formation of adapter dimers, thereby improving the recovery of miRNA sequences and enabling the construction of TGIRT-seq libraries from even smaller amounts of starting material. Additionally, using a miRNA reference set containing an equimolar mixture of 962 human miRNAs, we systematically analyzed 5′- and 3′-end biases in TGIRT-seq, and developed biochemical and computational methods for ameliorating these biases. We found that the 5′-sequence biases introduced mainly by the thermostable 5′ App RNA/DNA ligase could be computationally corrected and that 3′-biases introduced by TGIRT template-switching could be corrected either computationally or by employing an altered ratio of 3′-overhang nucleotides in the R2 RNA/R2R DNA primer mix.

## Results

### A single nucleotide change in the R2R adapter strongly decreases adapter dimer formation

Analysis of TGIRT-seq datasets obtained for fragmented UHRR or plasma DNA suggested that a major source of sequence bias is the DNA ligation step using the thermostable 5′ App DNA/RNA ligase, which has a preference for A or C and against U/T at position −3 from the 3′ end of the acceptor nucleic acid^7,28,29^. We noticed that the R2R adapter used previously for TGIRT-seq (denoted NTC based on its 3′ end sequence) has a C-residue at position −3 from its 3′ end, which favors the formation of R1R-R2R adapter dimers during the ligation step (Fig. 1B).

**Figure 2:**
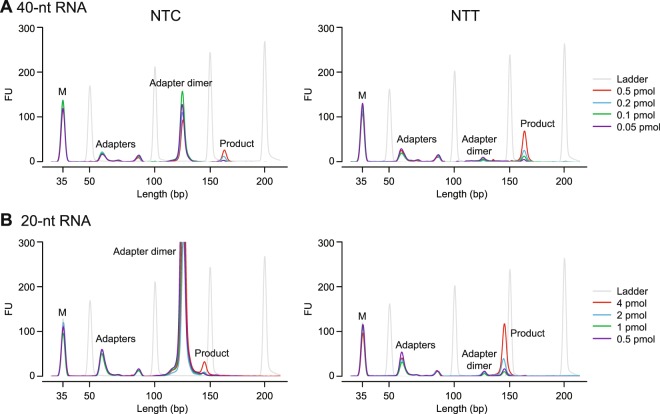
Bioanalyzer traces of TGIRT-seq libraries constructed from varying amounts of different-sized RNA oligonucleotides using either the NTC or NTT adapter. TGIRT-seq libraries were prepared from (**A**) 40-nt or (**B**) 20-nt RNA oligonucleotides using the workflow of Fig. 1A. After PCR for 12 cycles and one round of 1.4X AMPure beads clean-up, the libraries were analyzed on a 2100 Bioanalyzer (Agilent) using a high sensitivity DNA chip. M: internal markers.

To address this difficulty, we designed a new R2R adapter (denoted NTT) in which a single T residue was inserted at position −3, thereby replacing the favored C at this position with a disfavored T, but leaving the remainder of the R2R sequence unchanged (Fig. 1B). This internal nucleotide insertion required a complementary insertion in the R2 RNA oligonucleotide to maintain base pairing in the R2 RNA/R2R DNA heteroduplex. In a test reaction in which either the NTC or NTT R2R DNAs were ligated to R1R DNA followed by PCR with primers that add Illumina indices and capture sites as per the TGIRT-seq protocol (Fig. 1A), this single nucleotide change decreased the recovery of the R1R-R2R adapter dimers by 82–89% (n = 3; Fig. 1C). The lower level of adapter dimer formation enabled the construction of TGIRT-seq libraries with fewer rounds of AMPure beads clean-up and better recovery of library products corresponding to miRNAs and other very small RNAs. These improvements in turn enabled the construction of TGIRT-seq libraries from smaller amounts of starting material than with the NTC adapter (0.05 pmole of a 40-nt RNA and 0.5 pmole of a 20-nt RNA with 96–98% and 88–99% lower levels of adapter dimers, respectively, than the NTC adapter after 1 round of 1.4X AMPure beads clean-up; n = 3; Fig. 2).

### TGIRT-seq of ribo-depleted, fragmented UHRR with ERCC spike-ins using the modified R2R adapter

To assess the performance of the NTT R2R adapter, we used it for TGIRT-seq of ribo-depleted fragmented UHRR with ERCC spike-ins, as done previously for TGIRT-seq with the NTC adapter^7^. The TGIRT-seq libraries were constructed in triplicate with one round of 1.4X AMPure beads clean-up to remove adapter dimers and sequenced on an Illumina NextSeq 500 to obtain 61–105 million 75-nt paired-end reads (Supplementary Table S1). The read-pairs were mapped to a human genome reference sequence (Ensembl GRCh38 modified to include additional rRNA repeats) by using an updated TGIRT-seq mapping pipeline (see Methods). For comparison, raw sequencing reads from published TGIRT-seq datasets generated from similarly prepared fragmented UHRR samples using the NTC adapter^7^ were downloaded (NCBI SRA accession number SRP066009) and processed using the same bioinformatic pipeline.

**Figure 3:**
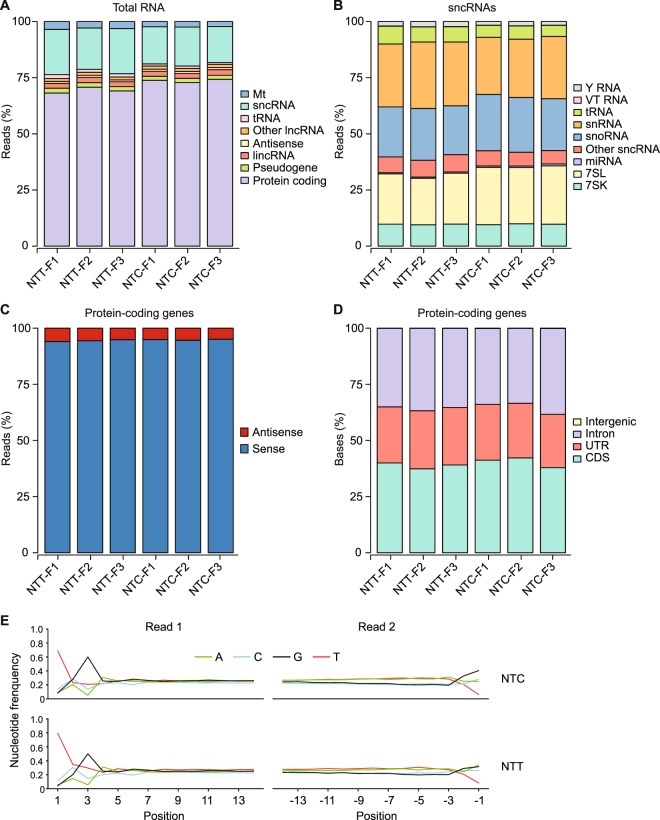
TGIRT-seq of ribo-depleted fragmented UHRR with ERCC spike-ins using the NTT and NTC adapters. TGIRT-seq libraries were prepared in triplicate for each adapter and sequenced on an Illumina NextSeq 500 to obtain 58–105 million 75-nt paired-end reads, which were mapped to a human reference genomic (Ensembl GRCh38) modified to include additional rRNA repeats (Methods and Supplementary Table S1). The datasets were used to generate stacked bar graphs showing the percentages of: (**A**) read-pairs that mapped concordantly in the annotated orientation to different categories of genomic features; (**B**) small ncRNA reads that mapped to different classes of small ncRNAs; (**C**) protein-coding gene reads that mapped to the sense or antisense strand; (**D**) bases in protein-coding gene reads that mapped to coding sequences (CDS), introns, 5′- and 3′-untranslated regions (UTRs), and intergenic regions. The name of the dataset is indicated below. (**E**) Aggregate nucleotide frequencies at the beginning of Read 1 (5′-RNA end; positions 1 to 14) and Read 2 (3′-RNA end; positions −1 to −14) in combined datasets for technical replicates obtained by TGIRT-seq of fragmented UHRR plus ERCC spike-ins with either the NTC or NTT adapter (datasets NTC-F1 to F3 and NTT-F1 to F3, respectively).

The datasets obtained using the NTT adapter had mapping rates similar to those for the NTC adapter (84–86% and 84–89%, respectively), with similar proportions of the mapped reads mapping concordantly in the correct orientation to annotated genomic features (92–94%; Supplementary Table S1). Scatter plots comparing the representation of RNAs in technical replicates obtained using the NTT and NTC adapters gave Spearman’s correlation coefficients (ρ) of 0.95–0.96 (Supplementary Fig. S1), and a histogram of the coefficients of variation of normalized counts from the replicates confirmed their similarly high reproducibility (94% and 92% of the protein-coding gene transcripts and spike-ins with normalized read count >10 have a coefficient of variation ≤25% for the NTT and NTC adapters, respectively, compared to 87% for TruSeq v3 in the benchmark datasets^30^ (Supplementary Fig. S2). Likewise, the normalized abundances (transcripts-per-million; TPM) of ERCC spike-ins from the TGIRT-seq datasets correlated well with the expected spike-ins inputs (ρ = 0.98; Supplementary Fig. S3). The datasets obtained using the NTT and NTC adapters showed no substantial differences in the profiles of reads mapping to different genomic features (Fig. 3A,B), the distribution of reads between the sense and antisense strands of protein-coding genes (Fig. 3C), or the proportions of bases mapping to different regions of protein-coding genes (Fig. 3D).

To assess sequence biases in the TGIRT-seq libraries, we plotted aggregate nucleotide frequencies as a function of position from the beginning of Read 1 and Read 2, respectively (Fig. 3E). The plots showed that the 5′- and 3′-end sequence biases are similar for the NTT and NTC adapters, with the 5′-RNA end bias for G or U and against A at position +3 being the reciprocal of the sequence preferences of the thermostable 5′ App DNA/RNA ligase for the 3′ end of the cDNA (see above), and the 3′-RNA end bias against U and for G at position −1 including a contribution from TGIRT template-switching. The apparent 5′ bias for U at position +1 at the 5′-RNA end includes a contribution from non-templated addition of an A residue to the 3′ end of cDNAs by the TGIRT enzyme. Additionally, both the 5′- and 3′-biases may include a contribution from the RNA fragmentation process^31^. Whatever the cause, nearly all of the sequence biases in TGIRT-seq libraries prepared from the fragmented UHRR were confined to the first 3 positions from the 5′ and 3′ ends of the RNA fragments, in contrast to Illumina TruSeq protocols, which also include substantial internal biases due to random hexamer priming^7,32^. Together, these results showed that the NTT adapter performs similarly to the previous NTC adapter for the analysis of ribo-depleted fragmented whole-cell RNAs, but requires fewer rounds of AMPure beads clean-up to remove adapter dimers (1 round for the NTT adapter compared to 3 rounds in the previous libraries obtained with the NTC adapter; Supplementary Table S1).

### TGIRT-seq of a miRNA reference set and analysis of 5′- of 3′-end biases

To evaluate the performance of the NTT adapter in miRNA sequencing, we used it to construct TGIRT-seq libraries of a miRNA reference set containing an equimolar mixture of 962 human miRNA sequences (miRXplore Universal Reference; Miltenyi Biotech) and compared its performance to that of the NTC adapter tested in parallel. Libraries prepared using each adapter were constructed in triplicate, with the libraries constructed using the NTT adapter requiring 1 round of 1.4X AMPure beads clean-up prior to sequencing compared to 4 rounds for those constructed using the NTC adapter (Supplementary Table S2). The libraries were sequenced on an Illumina NextSeq 500 to obtain 10–16 million 2 × 75-nt paired-end reads, which were mapped to the 962 reference miRNA sequences. The proportion of uniquely mapped reads was higher for the NTT adapter than the NTC adapter (86–88% and 63–74%, respectively), possibly reflecting that the multiple rounds of AMPure bead clean-up required for the NTC adapter resulted in differential loss of miRNA-sized products, whose sequences map uniquely to the miRNA reference sequences, compared to larger aberrant products resulting from multiple template switches, whose sequences map to multiple loci. Scatter plots comparing datasets for technical replicates gave ρ values of 1.00 between replicates with the same adapter and 0.94 between replicates with different adapters (Supplementary Fig. S4).

**Figure 4:**
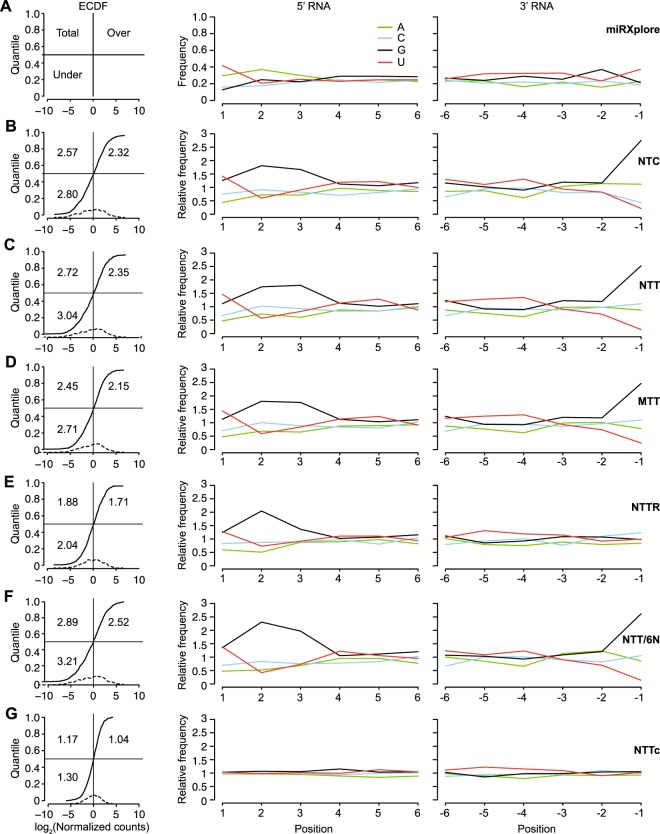
TGIRT-seq of the Miltenyi miRXplore miRNA reference set using the NTT or NTC adapters and comparison of different methods for mitigating 5′- and 3′-end biases. TGIRT-seq libraries were prepared from the Miltenyi miRXplore miRNA reference set containing 962 equimolar human miRNAs (Supplementary Table S2 and Methods). Datasets for each method (three combined datasets for NTC, NTT, MTT, and NTT and a single dataset for NTT/6N) were used to plot both the empirical cumulative distribution function (ECDF) of the log_2_ median-normalized counts for each miRNA ranked from least to most abundant (left panels), and the abundance-adjusted nucleotide frequencies at the 5′ end (positions +1 to +6) and 3′ end (positions −1 to −6) of the miRNA sequences in the dataset relative to those in the miRNA reference set (middle and right panels). Only uniquely mapped reads were counted. The numbers within the ECDF plots for each method indicate the root-mean-square error (RMSE) for over-represented miRNAs (top right), under-represented miRNAs (bottom left), and all miRNAs (top left). The curve plotted as a dashed line at the bottom of the ECDF plots indicates the distribution density of the 962 miRNAs in the dataset. (**A**) Miltenyi miRXplore reference set showing the ECDF plot layout (left panel) and the aggregate 5′- and 3′-nucleotide frequencies for all miRNAs in the Miltenyi miRXplore reference set assuming equimolar concentrations of the 962 miRNAs. (**B–G**) ECDF plots (left panels) and plots of the abundance-adjusted nucleotide frequencies at the 5′- and 3′- ends of miRNAs in TGIRT-seq datasets relative to those in the miRNA reference set (middle and right panels) for datasets obtained using (**B**) the NTC adapter; (**C**) the NTT adapter; (**D**) a modified NTT adapter mix in which the 3′ A overhang was replaced with a 3′ diaminopurine (denoted MTT); (**E**) a modified NTT adapter mix with an altered ratio of 3′ overhangs (A:C:G:T = 6.6:0.4:1:1; denoted NTTR); (**F**) the NTT adapter used in combination with an R1R adapter with six randomized nucleotides at its 5′ end (denoted NTT/6N); and (**G**) the NTT adapter after computational correction of 5′- and 3′-end biases (denoted NTTc).

To assess sampling biases of the miRNAs in the TGIRT-seq datasets, we combined the 3 technical replicates for each adapter and compared the representation of miRNAs in the combined datasets to that in the miRNA reference set (Fig. 4A–C). Plots of the empirical cumulative distribution function (ECDF) of the log_2_ normalized count for each miRNA in the reference set showed that the NTC and NTT adapters give similar representations of different miRNA species (RMSE = 2.57 and 2.72, respectively; Fig. 4A–C, left panels). Further, plots of either the abundance-adjusted nucleotide frequency as a function of position from the 5′ and 3′ ends of the miRNAs in the dataset relative to those in the reference set (Fig. 4A–C) or of the aggregate nucleotide frequency as a function of position from the beginning of Reads 1 and 2 (Supplementary Fig. S5; *cf*., with Fig. 3E for UHRR) showed that sequence bias is similar for the two adapters and largely confined to the first 3 nucleotides from the 5′ and 3′ ends of the miRNAs (Fig. 4A–C, middle and left panels).

Because the miRNAs in the reference set have known sequences, we could now more accurately assess the degree and cause of the sampling bias introduced by TGIRT-seq than could be done with fragmented whole-cell RNAs. The 5′ bias includes but is not limited to the known sequence preferences of the 5′ App DNA/RNA ligase (*e*.*g*., over-representation of G at position +3 of the RNA sequence compared to the reference set RNAs), while the 3′ bias due primarily to template-switching favors reference set miRNAs with a 3′ G residue and strongly disfavors miRNAs with a 3′ U residue (Fig. 4A–C, middle and right panels).

### Contribution of TGIRT-seq 5′- and 3′-end biases to miRNAs measurement errors

**Figure 5:**
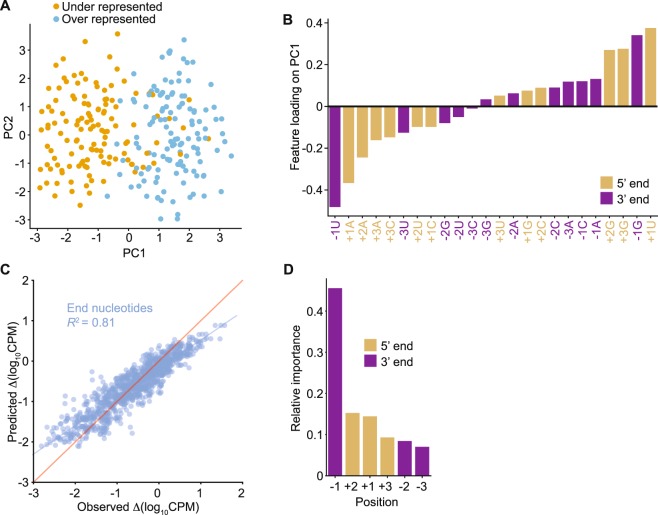
Effect of 5′- and 3′-end sequences on the representation of miRNAs in TGIRT-seq datasets. (**A**) Principal component analysis biplot for over- and under-represented miRNAs in TGIRT-seq of the Miltenyi miRXplore miRNA reference set in combined datasets for the three technical replicates obtained using the NTT adapter. The first three bases from the 5′- and 3′ ends of over- and under-represented miRNAs (defined as those whose log_2_CPM was at least one standard deviation higher or lower, respectively, than the mean log_2_CPM for all miRNAs in the reference set; Supplementary Fig. S6) were subject to principal component analysis. The first two principal components are shown. Each point indicates a miRNA, with over- and under-represented miRNAs colored as indicated in the Figure. (**B**) Relative importance of features of the first principal component. The fitted values from the first principal component are plotted for each base at each nucleotide position (feature) in ascending order. 5′- and 3′-end nucleotides are color coded as indicated in the Figure. (**C**) Random forest regression modeling of miRNA-seq quantification errors. A random forest regression model (*R*^2^ = 0.81) based on the first three 5′- and 3′-end positions was trained on the 962 miRNAs in the combined datasets for the 3 technical replicates obtained using the NTT adapter, and the predicted measurement errors (∆log_10_CPM predicted by the model) were plotted against the observed measurement errors (∆log_10_CPM obtained directly from sequencing data) for each miRNA. The blue line shows the fitted linear regression between the observed and predicted measurement errors, and the red line indicates hypothetical perfect prediction with slope = 1 and y-intercept = 0. (**D**) Relative importance of the position-specific preferences in TGIRT-seq. The relative importance of the 5′- and 3′-end positions from the random forest regression model were plotted in descending order. Each bar represents the relative importance of the indicated position color coded as indicated in the figure.

To quantify the contributions of TGIRT-seq 5′- and 3′-end biases to measurement errors for the miRNA reference set, we correlated the representations of each miRNA in the combined datasets obtained with the NTT adapter with its 5′- and 3′-end sequences. As our findings for both the fragmented UHRR (Fig. 3E) and the miRNA reference set (Fig. 4B,C middle and right panels and Supplementary Fig. S5) showed that much of the bias is confined to the first 3 nucleotides from each end of the RNA, we focused on these positions. For this analysis, we defined over- and under-represented miRNAs as those whose log_10_ Counts-Per-Million (CPM) values were ≥1 standard deviation higher and lower, respectively, than the mean log_10_ CPM for all of the miRNAs in the reference set (Supplementary Fig. S6). Principal component analysis (PCA) based on the first 3 nucleotides from the 5′ and 3′ ends of the miRNA showed that the over- and under-represented miRNAs were almost linearly separable along the first principal component (PC1) of the PCA biplot (Fig. 5A).

To identify the contribution of different nucleotides to the miRNA recovery rate, we inspected the factor loadings on PC1 (Fig. 5B). This showed that 3 of the top 4 contributing factors for over-represented miRNAs were from 5′ positions with the most favored bases being 5′ +1U; +3G and +2G (Fig. 5B, right side of plot). Moreover, 3 out of the top 4 contributing factors for under-represented miRNAs were also from the 5′ positions with the most disfavored bases being 5′ +1A, +2A and +3A (Fig. 5B, left side of plot). However, the largest contributor for under-represented miRNAs and second largest for over-represented miRNAs was the 3′ terminal nucleotide (position −1), which favored a G residue and disfavored a U residue (Fig. 5B). By fitting the data to a random forest regression model, we found that the position-specific nucleotide preferences at the first three nucleotides from the 5′ and 3′ ends of the miRNA account for 81% (*R*^2^ = 0.81) of the measurement errors (Fig. 5C). A k-fold cross-validation test of the random forest regression model in which the 962 miRNAs were divided into 8 subgroups, each of which was tested with a model trained on the remaining subgroup, gave *R*^2^ values of 0.46 to 0.66 (Supplementary Fig. S7A). By contrast, a model trained similarly using internal positions +4 to +6 and −4 to −6 performed poorly (*R*^2^ = −0.06 to 0.06; Supplementary Fig. S7B), confirming the importance of the first three 5′- and 3′-end positions compared to the internal positions.

As the random forest regression model predicts the full spectrum of miRNA measurement errors, we could use it to quantitatively assess the contributions of each of the first three 5′- and 3′-end positions to the measurement errors (Fig. 5D). The results were generally consistent with the PCA, which identified nucleotide combinations that separate over- and under-represented miRNAs. Thus, the −1 position was identified as having the greatest contribution to the bias, followed by the +2, +1 and +3 positions (Fig. 5D and Supplementary Fig. S7). A simple calculation summing the relative importance of the positions suggested that the 5′- and 3′-end biases contributed 40% and 60%, respectively, of the measurement errors due to end biases. However, we also found that nucleotides at some 5′- and 3′-end positions of the miRNAs in the reference set are correlated, in some cases with χ^2^-test -log_10_ p-values > 10 (*e*.*g*., 42% of the miRNAs with a disfavored A at position +3 have a disfavored U at position −1; Supplementary Fig. S8). This correlation raises the possibility that some of the apparent bias at 3′-end position −1 may reflect the 5′-adapter-ligation bias rather than the template-switching bias, consistent with the lower 3′-end bias seen previously in TGIRT-seq of a miRNA reference set using CircLigase instead of the 5′ App DNA/RNA ligase^6^ (and see below).

### Biochemical and computational methods for remediating 5′- and 3′-biases in TGIRT-seq

Having investigated the sources of the 5′- and 3′-end bias in the TGIRT-seq protocol, we next explored biochemical and computational approaches for mitigating these biases. For the 3′ bias, we first thought that the preference for a G residue and against a U residue at position −1 might reflect the strength of the base-pairing interaction between that nucleotide and the 3′-overhang nucleotide of the DNA primer that is used to direct TGIRT template-switching, with a strong rG/dC base pair favored over a weak rU/dA pair. However, changing the 3′-A overhang in the NTT primer mix to a diaminopurine (denoted MTT) to enable a stronger base pair with 3 instead of 2 H-bonds to a 3′ U only slightly ameliorated this bias (RMSE decreased from 2.72 to 2.45; Fig. 4C,D). Although using a locked nucleic acid for the 3′-overhang A might further ameliorate this bias, it would also increase cost, and the lack of bias for the reciprocal combinations (rA/dU) and (rC/dG) (Fig. 4C) suggests that the 3′ biases against U and for G are due largely to nucleotide preferences of the TGIRT enzyme.

**Figure 6:**
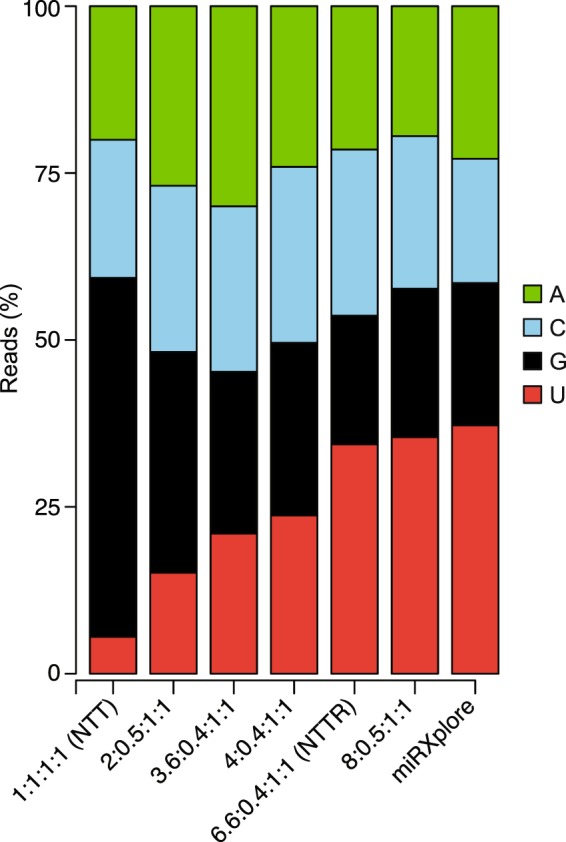
TGIRT-seq of the Miltenyi miRXplore miRNA reference set using R2 RNA/R2R DNA adapters with different ratios of the 3′-DNA overhang nucleotides. The stacked bar graphs show the percentages of miRNAs having A, C, G, and U 3′-end nucleotides, color coded as indicated in the Figure, in the datasets obtained with different ratios of 3′-overhang nucleotides. The expected ratio in the miRNA reference set is shown by the bar graph at the right. Only uniquely mapped reads were counted.

Thus, we tried an alternate approach based on the previous finding that increasing or decreasing the proportion of a 3′-overhang nucleotide in the primer mix correspondingly increases or decreases the recovery of miRNAs having a complementary 3′ end in the TGIRT-seq libraries^6^. We extended this finding by constructing TGIRT-seq libraries from the miRNA reference set with a series of R2 RNA/R2R DNA adapter mixes with higher proportions of 3′ A overhangs and lower proportions of 3′ C overhangs (Fig. 6) and found that we could almost completely eliminate the 3′ bias in TGIRT-seq of the miRNA reference set by using a primer mix with a ratio of 3′ overhang nucleotides A:C:G:T of 6.6:0.4:1:1 (denoted NTTR; RMSE = 1.88; Figs 4E and 6, compare NTTR to bar graph on the right showing the proportion of 3′ nucleotides in the miRNA reference set).

For the 5′-ligation bias, we noted that established small RNA-seq methods that employ T4 RNA ligases I and II to sequentially ligate adapters to the 5′ and 3′ ends of RNAs or cDNAs benefit from employing DNA adapters with four randomized nucleotides at the ligated ends (referred to as 4N protocols), with such adapters giving lower bias and better coverage at low sequencing depths than those with invariant sequences at their ends^33–36^. However, miRNA libraries prepared by TGIRT-seq with an R1R adapter containing six randomized nucleotides at its 5′ end (denoted NTT/6N) did not decrease the ligation bias (RMSE = 2.89 compared to 2.72 for NTT with the R1R adapter without randomized nucleotides; Fig. 4F compared to Fig. 4C). This result may reflect that the ligation bias in methods that benefit from 4N adapters results largely from miRNA/adapter base-pairing interactions (referred to as “co-folding”), which can be ameliorated by providing adapters with randomized bases at the ligating end^34–37^. By contrast, because TGIRT-seq employs a thermostable ligase for a single-stranded ligation of a DNA adapter to a cDNA at high temperature, any bias resulting from base-pairing interactions between the adapter and acceptor cDNA may already be minimal.

As an alternative for addressing the sampling biases in TGIRT-seq, we built a proof-of-concept bias corrector for TGIRT miRNA-seq using the random forest regression model described above (Fig. 5C,D) to correct for the measurement errors due to 5′- and 3′-end biases. The bias corrector uses the first and last 3 nucleotides of each miRNA to predict the measurement errors, so that a corrected abundance can be computed by subtracting the predicted measurement error from the experimentally determined abundance of each miRNA (see Methods). By employing this computational correction on the TGIRT-seq datasets obtained using the NTT adapter (denoted NTTc), both the 5′- and 3′-end biases were largely corrected, and the aggregate frequencies of miRNA 5′- and 3′-end nucleotides in the dataset closely approached those of the miRNAs in the reference set (RMSE = 1.17; Fig. 4G). An alternative computational approach for 5′- and 3′-end bias correction, using a reweighting scheme based on the trinucleotide frequencies at the beginning of Reads 1 and 2^32^, did not ameliorate end biases nearly as well as the random forest regression model (Supplementary Fig. S9).

### Comparison of TGIRT-seq to other miRNA sequencing methods

**Figure 7:**
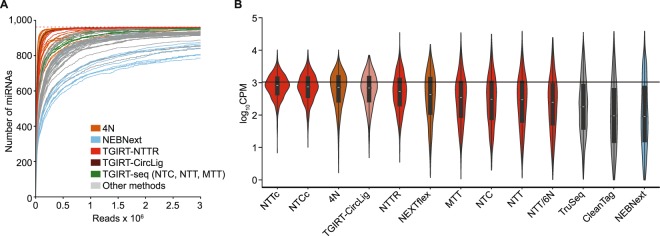
Saturation curves and differences in coverage for the 962 miRNAs in the Miltenyi miRXplore miRNA reference set for TGIRT-seq with or without different bias correction compared to published datasets for established small RNA-seq methods. For published datasets containing additional miRNAs, *in silico* subsamples containing only the 962 reference set miRNAs were used for the comparisons. (**A**) RNA-seq saturation curves. The curves show the number of reference set miRNAs with at least 10 reads at bins of 200 reads. As additional reads were included, the number of miRNAs with at least 10 reads increased. Curves were truncated at 3 million reads. The dotted red line at the top indicates the number of miRNAs in the Miltenyi miRXplore reference set. Each curve represents combined datasets, color-coded by the sequencing method as shown in the Figure for the best (4N ligation/NEXTflex; n = 24) and worst (NEBNext; n = 12) methods from the comparison of Giraldez *et al*.^36^, as well as TGIRT-seq (n = 3 for libraries prepared with the NTT, MTT, and NTC adapters), TGIRT-seq with the NTTR adapter (n = 3), TGIRT-seq with the NTT adapter and an R1R adapter containing six randomized 5′-end positions (NTT/6N; n = 1), and the TGIRT-CircLigase method (n = 1; Mohr *et al*.^6^). Other library preparation methods (gray lines) include NEBNext, TruSeq and CleanTag. (**B**) Violin plots of miRNA abundance in datasets obtained by different methods. The plots show the distribution of log_10_CPM for each miRNA in the reference set for each library preparation method (miRNA count = 2,886 for NTTc, 2,885 for NTCc, 23,088 for 4N ligation, 961 for TGIRT-CircLigase, 2,886 for NTTR, 5,522 for NEXTflex, 2,886 for MTT, 2,886 for NTC, 2,886 for NTT, 962 for NTT/6N, 30,757 for TruSeq, 3,815 for CleanTag, and 11,452 for NEBNext). NTTc and NTCc denote TGIRT-seq datasets obtained using the NTT or NTC adapters that were computationally corrected using the random forest regression model trained with the combined NTT datasets (Fig. 5C,D). The black horizontal line indicates the expected CPM values (CPM = 1,039.5) for each miRNA for a uniform distribution of 1,000,000 reads to 962 miRNAs (*i*.*e*., unbiased sampling for each miRNA). The library preparation and correction methods are ordered from the lowest to highest deviation between the median CPM (white point within the violin) and the expected CPM. The black boxes in the violins indicate the interval between first and third quartiles, and the vertical lines indicate the 95% confidence interval for each method.

Figure 7 compares TGIRT-seq of the miRNA reference set using different methods of bias correction described above with published datasets obtained by using established small RNA library preparation methods on RNA samples containing the 962 miRNAs in the Miltenyi miRXplore reference set. Because some of the published datasets contain additional miRNAs, we created *in silico* subsamples containing only the 962 reference set miRNAs from each dataset for these comparisons. Figure 7A shows saturation curves (*i*.*e*., plots of the recovery of miRNAs with a read count of ≥10 as a function of sequencing depth), and Fig. 7B shows violin plots of the log_10_CPM values for the reference set miRNAs obtained by the different methods. The plots confirmed the previous finding^36^ that the 4N protocols perform better than other small RNA-seq methods both in sampling miRNAs (reaching the plateau at smaller library sizes; Fig. 7A) and in obtaining expected log_10_CPM values (median closest to the red line) with smaller variance across the measured miRNA CPM values (shorter distance between the two ends of the violin plot; Fig. 7B). TGIRT-seq with NTTR adapter performed almost as well as the 4N protocols and better than TGIRT-seq with other adapters in the recovery of miRNA sequences as a function of read depth, reflecting that the altered ratio of R2R adapter 3′ overhangs improves the recovery of miRNAs with disfavored 3′-end sequences (Fig. 7A). Further, TGIRT-seq with the NTT or NTC adapters with computational correction (denoted NTTc and NTCc, respectively) performed slightly better than the 4N protocols in overall sampling bias and variance, and substantially better than commercial small RNA sequencing methods, including NEXTflex, TruSeq, CleanTag, and NEBNext (Fig. 7B). Based on a previously published dataset (SRA accession number SRR833775^6^), the TGIRT CircLigase method, employing TGRT template-switching by TeI4c RT instead of GsI-IIC RT and a cDNA gel-purification step prior to circularization, performed about as well as the 4N protocols both in miRNA recovery as a function of sequencing depth and in overall sample bias and variance (Fig. 7A,B), in agreement with previous findings^6^.

### Factors other than end biases that may contribute to measurement errors in TGIRT-seq

**Figure 8:**
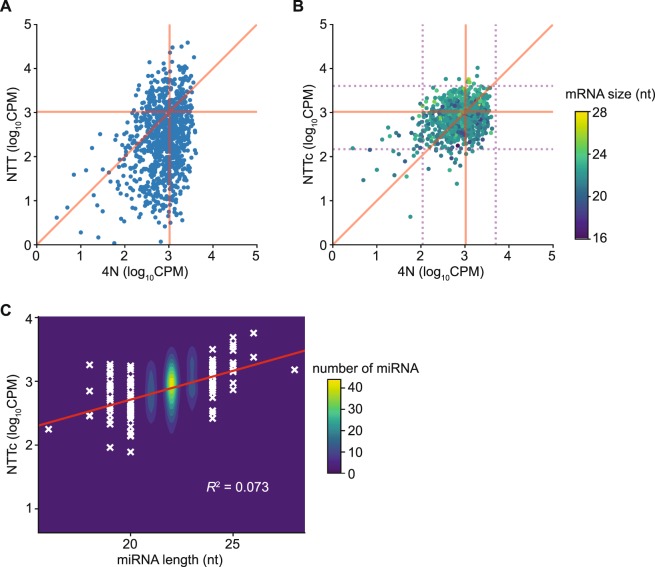
Representation of the Miltenyi miRXplore miRNA reference set in datasets obtained by TGIRT-seq with the NTT adapter before and after computational correction compared to representation of the same miRNAs in datasets obtained using 4N protocols. (**A**) miRNA representation for TGIRT-seq NTT versus 4N. Log_10_CPM values for each miRNA in combined TGIRT-seq NTT datasets (n = 3) are plotted against those in combined datasets for 4N protocols (n = 24; Gilardez *et al*.^36^). Each point represents one miRNA. (**B**) The same comparison as (A) after computational correction of the TGIRT-seq NTT dataset using the random forest regression model (Fig. 5C,D). In (B), miRNAs are color-coded by their lengths (scale to the right). The purple dotted lines delineate 95% confidence intervals (2 standard deviations from the mean) of the miRNAs for 4N (vertical dotted lines) or NTT (horizontal dotted lines). The box formed by the intersections of the dotted lines encompasses 892 miRNAs that lie within these confidence intervals and were used for comparison with over- and under-represented miRNAs in Fig. 9. The expected CPM values (CPM = 1,039.5 for each of the 962 equimolar miRNAs) are indicated by horizontal and vertical orange lines for TGIRT-seq and the 4N protocols, respectively. The diagonal red line indicates cases where the CPM values from NTT are equal to those for 4N protocols. (**C**) Correlation between miRNA abundances and miRNA length. Two-dimensional kernel density estimation of the distribution for miRNA abundances and lengths (n = 962) is shown. The linear regression, with the equation: log_10_CPM = 0.09 (miRNA size) +0.9, is plotted as a red line, and miRNAs with length <21 or >23 nt are indicated as white crosses. The coefficient of determinant (*R*^2^) is indicated in the plot. The color scale indicates the numbers of miRNAs not shown as crosses.

To further investigate sources of bias in miRNA sequencing, we compared the over- and under-represented reference set miRNAs in datasets obtained by TGIRT-seq NTT and 4N ligation protocols (Fig. 8). In agreement with the findings above, we found that most of the under- and over-represented miRNAs in TGIRT-seq compared to 4N protocols were due to 5′- and 3′-end sequence biases that could be substantially corrected computationally, so that the abundance of most of the reference miRNAs after correction was similar to that in 4N protocols (Fig. 8A,B). However, a small number of miRNAs remained substantially under- or over-represented in both TGIRT-seq and 4N protocols.

To identify other factors that might have contributed to the biased representation of these outlier miRNAs in TGIRT-seq, we defined over- and under-represented miRNAs as those whose log_10_CPM values after computational correction for end biases were ≥2 standard deviations higher (n = 8) or lower (n = 27) than the mean log_10_CPM, and then compared several potentially bias-inducing characteristics of these miRNAs to the remaining 927 more uniformly represented miRNAs (those in the center box in Fig. 8B). The compared characteristics included miRNA length, GC content, stability of potential secondary structure (self-fold free energy), potential of the R1R adapter to co-fold with the miRNA cDNA ligated in the second step of TGIRT-seq (co-fold free energy), and the numbers of unpaired (*i*.*e*., free) 5′ and 3′ nucleotides in the most stable predicted secondary structure.

**Figure 9:**
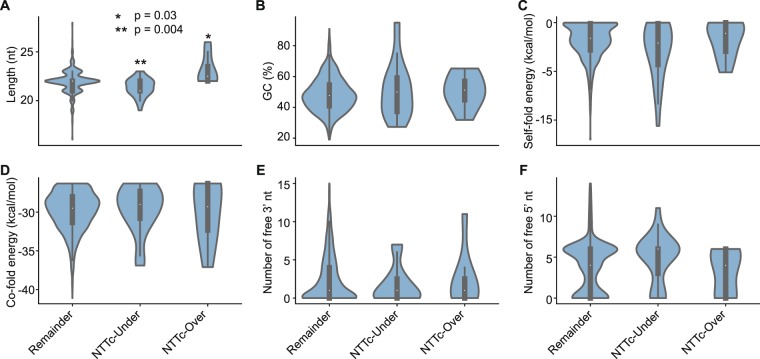
Factors other than end biases that may contribute to measurement errors in miRNA representation in TGIRT-seq. The figure shows violin plots comparing several potentially bias-inducing characteristics in over-represented (n = 8) or under-represented miRNAs (n = 27) in combined TGIRT-seq datasets obtained using the NTT adapter defined as those with log_10_CPM values two or more standard deviations higher than the mean log_10_CPM compared to the remaining 927 miRNAs (those within the center box in Fig. 8B). The characteristics compared include: (**A**) miRNA length; (**B**) GC content; (**C**) the minimum free energy of the most stable predicted secondary structure (self-fold energy) computed by the Vienna RNA package; (**D**) the predicted minimum free energy of base pairing between the R1R adapter and the miRNA cDNA with attached R2R adapter to which it is ligated in the second step of TGIRT-seq (Fig. 1A) computed by Vienna RNA package (co-fold energy); (**E**) the number of unpaired (free) 3′ nucleotides in the predicted secondary structure; and (**F**) the number of unpaired (free) 5′ nucleotides in the predicted secondary structure. Asterisks on the top of the violins indicate significance of the difference between the outliers and remaining miRNAs determined by Wilcoxon test (*p = 0.03; **p = 0.004).

Violin plots of the distribution of miRNAs in each of the above group as a function of the compared characteristic showed that miRNA length is the only tested factor that contributes significantly to the under- or over-representation of these outlier miRNAs in TGIRT-seq (Wilcoxon test p-values = 0.004 and 0.03, respectively; Fig. 9). However, for the larger group of 962 miRNAs, a plot of miRNA representation as a function of length showed only a weak correlation (*R*^2^ = 0.073; Fig. 8C). The Violin plots confirmed that neither self-folding of the miRNAs nor co-folding of the R1R adapter with the miRNA cDNAs contributed significantly to the under-representation of the outlier miRNAs in TGIRT-seq (Fig. 9C,D).

## Discussion

By avoiding gel-purification steps, the TGIRT Total RNA-seq method enables the rapid construction of comprehensive RNA-seq libraries containing nearly all RNA biotypes from small amounts of starting materials with less overall bias than other transcriptome-profiling methods^7,8,14^. Here, we addressed two issues in TGIRT-seq library preparation, the disproportionate loss of miRNA sequences during AMPure beads clean-up of adapter dimers, and sampling biases resulting from 5′- and 3′-end sequences preferences in the ssDNA ligation and TGIRT template-switching steps.

First, to address the adapter dimer problem, we used the known sequence biases of the thermostable 5′ App DNA/RNA ligase employed for R1R adapter ligation to design an R2R adapter with a single nucleotide change that strongly decreases adapter dimer formation during TGIRT-seq library preparation (88–99% lower compared to the previous NTC adapter; Fig. 2). The redesigned R2R adapter (denoted NTT) decreases the number of rounds of AMPure beads clean-up required to remove adapter dimers, thereby increasing the recovery of very small RNAs and enabling the construction of TGIRT-seq libraries from smaller amounts of starting materials.

A previous approach for decreasing adapter dimer formation in RNA-seq protocols in which DNA adapters are ligated to both 5′- and 3′-RNA ends uses adapters with chemical modifications near the ligated ends of both adapters^38^. These chemical modifications were hypothesized to inhibit ligation and impede subsequent reverse transcription when brought into close proximity in adapter dimers, but not when separated by a library insert. The authors carefully noted, however, that adapter dimer suppression was largely dependent upon the sequence of the adapters and that the same chemical modifications did not achieve the same degree of suppression with other adapter sequences^38^. Our results extend these findings by showing that, at least for some ligases, small changes in adapter sequences based on analysis of the sequence preferences of the ligase is sufficient to strongly suppress adapter dimer formation without resorting to chemical modifications.

Next, we used TGIRT-seq of miRNA reference sets to analyze and correct 5′- and 3′-end biases in miRNA-seq. The 5′-end bias in TGIRT-seq is due in large part to sequence biases of the thermostable 5′ App DNA/RNA ligase used for single-stranded ligation of the R1R adapter to the 3′ end of the cDNA (Fig. 1A). We found that this bias could not be mitigated by using an R1R adapter with randomized nucleotides near its 5′ end, as in 4N ligation RNA-seq protocols, but could be corrected computationally by using a random forest regression model to give the same level of bias as in 4N protocols. The 3′-end bias in TGIRT-seq is confined largely to the 3′ nucleotide of the target miRNA, which base pairs with the 3′ overhang of the DNA primer mix during template-switching. We first thought that this 3′ bias for G and against U might reflect the relative strengths of the rG/dC and rU/dA base pair between the 3′ nucleotide of the miRNA and the 3′-overhang nucleotide of the R2R DNA primer. However, this 3′ bias could be only slightly ameliorated by substituting a diaminopurine 3′ overhang to enable a stronger base pair to a 3′ U and was not observed for the reciprocal combinations rC/dG and rA/dU, suggesting that it results from nucleotide sequence preferences of the TGIRT enzyme. In a test with miRNA reference set, this 3′ bias could be almost completely remediated either by using primer mixes with an adjusted ratio of 3′ A, C, G, and T overhang nucleotides to compensate for the sequence preferences of the TGIRT enzyme or computationally by using the random forest regression algorithm, which simultaneously corrects the 5′ bias (Fig. 4). The degree of computational correction that can be attained for TGIRT-seq is possible because sequences biases are almost entirely confined to the first three nucleotides from either end of the RNA template.

Although the computational corrections for 5′- and 3′-end biases in TGIRT-seq and 4N ligation RNA-seq protocols address different factors, sequence bias in TGIRT-seq and adapter/miRNA co-folding in the 4N protocols^33–36^, they achieve very similar degrees of overall correction in the datasets for miRNA reference sets, with relatively few outliers that are differentially corrected by one or the other method (Fig. 8). This likely reflects that the biases corrected by the two methods are orthogonal. The TGIRT-seq correction for 5′-end bias addresses sequence preferences of the ligase, which are larger for the 5′ App RNA/DNA ligase than for the T4 RNA ligases used in the 4N protocols^33–37^, while the 4N correction addresses adapter/miRNA co-folding, which is not a significant factor in the high temperature ssDNA ligation in TGIRT-seq (Fig. 9). Examination of outlier miRNAs after correction for TGIRT-seq. 5′- and 3′-end biases indicates that miRNA length may be a contributing factor for under- and over-representation of some but not most miRNAs (Fig. 9).

As noted previously by Giraldez *et al*.^36^, biological samples would likely behave differently from synthetic RNA pools tested at a single concentration *in vitro*. Thus, although the random forest regression model described here provides insight into the sources of bias and proof-of-concept that this method can be used for bias correction in RNA-seq, its use for biological samples requires parallel validation in different sample types (*e*.*g*., by heterologous miRNA spikes-ins added at different concentrations) and/or confirmation by orthogonal approaches, such as RT-qPCR, microarrays, or bead-based hybridization assays (*e*.*g*., Firefly; Abcam)^39–42^. Longer term, the bias in 5′-adapter addition might be addressed by using a modified or different ligase, but a less biased ligase might also lead to increased production of adapter dimers. Thus, a preferable approach may be to use an alternative method for 5′-adapter addition, such as leveraging the ability of TGIRT-III to add non-templated A residues to the 3′ of cDNAs to enable template-switching to an acceptor oligonucleotide with 3′ U residues, analogous to the Clontech/Takara SMART-seq protocols^43,44^. The 3′ bias in TGIRT template-switching seems less problematic as it can be addressed either computationally or by using an adjusted ratio of 3′ overhangs in R2 RNA/R2R DNA starter duplex. Longer term, it might also be addressed by using a different or modified TGIRT enzyme with less sequence bias. The recently determined crystal structure of full-length GsI-IIC RT in an active conformation with bound substrates^12^ provides a platform for detailed analysis of the structural basis and possible alleviation of this 3′-end bias.

An attractive feature of the TGIRT Total RNA-seq method is that it can comprehensively profile different RNA size classes in a single RNA-seq experiment, enabling applications such as correlation of mRNA codon usages with isodecoder tRNA levels^45,46^; comparison of expression levels of small ncRNAs and mRNAs encoding components of the same RNP complexes^14^; and the analysis of tRNAs and tRNA fragments or mature, pre-, and pri-miRNA in the same RNA-seq^7,8,23,47^. Previous work showed that the total RNA-seq protocol with TGIRT-III works well for quantitation of small RNAs down to ~60 nt^14^, and the use of the new NTT adapter substantially improves the recovery of miRNAs sequences, both for the miRNA reference sets used here and in cellular, exosomal, and human plasma RNA samples analyzed in our laboratory. We note, however, that even with the NTT adapters, the recovery of miRNA sequences in the TGIRT Total RNA-seq method with GsI-IIC RT (TGIRT-III) is less efficient than that of larger RNAs (Fig. 2), reflecting that miRNA library products may still be differentially lost at clean-up steps in the TGIRT-seq protocol (including the single round of Ampure beads clean-up to remove PCR primers and residual adapter dimers) and that larger RNAs out compete very small RNAs (<60 nt) for reverse transcription by GsI-IIC RT in mixed-sized RNA preparations^8^. For studies focused on mature miRNAs, the latter issue could be minimized by introducing a size-selection step to obtain more uniformly sized RNA preparations and/or by employing orthogonal approaches (see above) to confirm quantitative inferences about miRNA abundance. Additionally, based on comparison of published datasets, we find that alternative TGIRT-CircLigase method, which includes a gel-purification step, performed similarly to 4N protocols in both the recovery of miRNA sequences as a function of sequencing depth and overall variance from the expected CPM values (Fig. 7), and at present, it may be the TGIRT method of choice for studies focused primarily on mature miRNAs. We also note that another TGIRT enzyme, TeI4c RT, which has so far not been used extensively for RNA-seq, has significantly different properties than GsI-IIC RT, including the ability to synthesize even longer cDNAs and to give a more uniform representation of RNAs <60 nt in mixed-sized RNA preparations^8^. The numerous group II intron RTs identified by the sequencing of bacterial, archaeal, and organellar genomes may provide a rich resource for the identification of enzymes with even more beneficial properties for RNA-seq than those tested thus far.

## Methods

### DNA and RNA oligonucleotides

The sequences of DNA and RNA oligonucleotides used in this work are summarized in Supplementary Table S3. All oligonucleotides were purchased from Integrated DNA Technologies (IDT) in RNase-free HPLC-purified form. R2R oligonucleotides with different 3′ nucleotides were hand-mixed prior to annealing to the R2 RNA oligonucleotide to obtain the desired ratio of single nucleotide 3′-overhangs^7,8^. The NTT and NTC primer mixes contain an equimolar mix of R2R DNAs with 3′ A, C, G, and T residues. In the MTT primer mix, the R2R DNA with a 3′ A was replaced with a 3′ diaminopurine. In the NTTR primer mix, R2R DNAs with 3′ A, C, G, and T were mixed at a ratio of 6.6:0.4:1:1. Primer mixes with other ratios of 3′ nucleotides described in Results (Fig. 6) were prepared similarly.

### RNA preparations

The miRXplore miRNA reference set was purchased from Miltenyi Biotech. The RNA was dissolved in nuclease-free water (Invitrogen), adjusted to 1 μM, and aliquoted for storage at −80 °C. Fragmented human reference RNA samples were prepared as described^7^. 50 μl of Universal Human Reference RNA (UHRR; Agilent) at 1 μg/μl was mixed with 1 μl of ERCC ExFold Mix 1 (Thermo Fisher Scientific; denoted ERCC spike-ins) prepared according to the provided protocol. 2 μl of the resulting UHRR sample with ERCC spike-ins was ribo-depleted by using a Human/Mouse/Rat Ribo-zero rRNA removal kit (Illumina), fragmented to 70–100 nt by using an NEBNext Magnesium RNA Fragmentation Module (94 °C for 7 min; New England Biolabs), and treated with T4 polynucleotide kinase (Epicentre) to remove 3′ phosphates that impede TGIRT template-switching^7^. After each of the above steps, the RNA was cleaned-up by using a Zymo RNA Clean & Concentrator kit, with 8 volumes of ethanol added to the input RNA to maximize the recovery of small RNAs^7^. The fragment size range and RNA concentration were verified by using a 2100 Bioanalyzer (Agilent) with an Agilent 6000 RNA pico chip and aliquoted into 6 ng/3 μl portions for storage in −80 °C.

### TGIRT-seq

TGIRT-seq libraries were prepared as described^7,8^ using 6 ng of fragmented Universal Human Reference RNA (UHRR) with ERCC spike-ins or 50 nM Miltenyi miRXplore RNA prepared as described above. The template-switching and reverse transcription reactions were done as described^7,8^ with 1 μM TGIRT-III (InGex) and 100 nM pre-annealed R2 RNA/R2R DNA in 20 μl of reaction medium containing 450 mM NaCl, 5 mM MgCl_2_, 20 mM Tris-HCl, 5 mM DTT, pH 7.5. Reactions were set up with all components except dNTPs, pre-incubated for 30 min at room temperature, a step that increases the efficiency of template-switching and reverse transcription, and then initiated by adding dNTPs (final concentrations 1 mM each of dATP, dCTP, dGTP, and dTTP). The template-switching reactions were incubated for 15 min at 60 °C and then terminated by adding 1 μl 5 M NaOH to degrade RNA and heating at 95 °C for 5 min followed by neutralization with 1 μl 5 M HCl and two rounds of MinElute column clean-up (Qiagen) to decrease the amount of unused R2R DNA adapter. The R1R DNA adapter was pre-adenylated by using an adenylation kit (New England Biolabs) and then ligated to the 3′ end of the cDNA by using thermostable 5′ App DNA/RNA Ligase (New England Biolabs) for 2 h at 65 °C. The ligated products were purified by using a MinElute Reaction Cleanup Kit and amplified by PCR with Phusion High-Fidelity DNA polymerase (Thermo Fisher Scientific; denaturation at 98 °C for 5 sec followed by 12 cycles of 98 °C 5 sec, 60 °C 10 sec, 72 °C 15 sec and then held at 4 °C). The PCR products were cleaned up by using Agencourt AMPure XP beads (1.4X volume; Beckman Coulter) and sequenced on an Illumina NextSeq 500 instrument to obtain 2 × 75-nt paired-end reads.

### Bioinformatic analysis

Datasets obtained for ribo-depleted, fragmented UHRR plus ERCC spike-ins using the NTT adapter were compared with published datasets for identically prepared samples using the NTC adapter downloaded from NCBI (SRA accession number SRP066009^7^). After removing the extra T residue introduced by the NTT adapter at the 5′ end of Read 2, reads from datasets obtained by using the NTT and NTC adapters were trimmed with cutadapt^48^ 1.16 to remove Illumina TruSeq adapters and PCR primer sequences (sequencing quality score cut-off at 20), and reads <15-nt after trimming were discarded. Reads were then mapped by using HISAT2^49^ v2.0.2 with default settings to a human genome reference sequence (Ensembl GRCh38 Release 76) combined with additional contigs for 5S and 45S rRNA genes and the *E*. *coli* genome sequence (Genebank: NC_000913) (denoted Pass 1). The additional contigs for the 5S and 45S rRNA genes included the 2.2-kb 5S rRNA repeats from the 5S rRNA cluster on chromosome 1 (1q42, GeneBank: X12811) and the 43-kb 45S rRNA repeats that contained 5.8S, 18S and 28S rRNAs from clusters on chromosomes 13, 14, 15, 21, and 22 (GeneBank: U13369). Unmapped reads from Pass 1 were re-mapped to Ensembl GRCh38 Release 76 by Bowtie 2^50^ v2.2.6 with local alignment to improve the mapping rate for reads containing post-transcriptionally added 5′ or 3′ nucleotides (*e*.*g*., CCA and poly(U)), short untrimmed adapter sequences, or non-templated nucleotides added to the 3′ end of the cDNAs by the TGIRT enzyme (denoted Pass 2). The uniquely mapped reads from Passes 1 and 2 were combined using Samtools^51^ v1.8. To process multiply mapped reads, we examined different alignments with the highest mapping score and selected the alignment with the shortest distance between the two paired ends (*i*.*e*., the shortest read span). In the case of ties between reads mapping to rRNA and non-rRNA sequences, the read was assigned to the rRNA sequence, and in other cases, the read was assigned randomly to one of the tied choices. Uniquely mapped reads and the filtered multiply mapped reads were combined and intersected with gene annotations (Ensembl GRCh38 Release 76) supplemented with the RNY5 gene and its 10 pseudogene sequences, which were not annotated in this release, to generate the counts for individual features. Coverage of each feature was calculated by Bedtools^52^. To avoid miscounting of embedded sncRNAs, the reads were first intersected with sncRNA annotations, and the remaining reads were then intersected with the annotations for protein-coding genes, lincRNAs, antisense, and other lncRNAs. To further improve the mapping rate for tRNAs and rRNAs, we combined reads that were uniquely or multiply mapped to tRNAs or rRNAs in the initial alignments and re-mapped them to tRNA (Genomic tRNA Database and UCSC genome browser website) or rRNA (GeneBank: X12811 and U13369) reference sequences using Bowtie 2 local alignment.

For correlation analysis, RNA-seq datasets were normalized for the total number of mapped reads by using DESeq2^53^ and plotted in R. Reads that mapped to protein-coding genes were analyzed by Picard (http://broadinstitute.github.io/picard/) to calculate the percentage of bases in CDS, UTR, intron, and intergenic regions.

For datasets obtained for the Miltenyi miRXplore miRNA reference set, reads obtained using the NTT and NTC adapters were processed as described above for UHRR datasets and then mapped with Bowtie2 using local alignment with default settings to the Miltenyi miRXplore reference sequences. Uniquely mapped reads with lengths between 15 and 40 nt (86–88% of the mapped reads for the NTT adapter; Supplementary Table S2) were retrieved and used to calculate the counts table for 962 miRNAs. Counts from each dataset were median normalized, log_2_ transformed, and used to generate scatter plots, empirical cumulative distribution function (ECDF) plots, and nucleotide frequency plots in R. RMSE was calculated using log_2_ transformed median normalized counts.

### Correction of 5′- and 3′-end biases

miRNA sequence biases were analyzed with customized scripts using pysam^51^ and SciPy ecosystem (http://www.scipy.org/). The predicted deviations between the expected log_10_ miRNA abundance for each miRNA (log_10_CPM; 962 equilmolar miRNAs from Miltenyi miRXplore reference set) and measured log_10_ abundance for that miRNA were calculated by using a random forest regression model implemented in R^54^ according to the following equation:

$${\rm{\Delta }}logCP{M}_{m}=f({x}_{m,1},{x}_{m,2},{x}_{m,3},{x}_{m,-3},{x}_{m,-2},{x}_{m,-1})$$

where ${\rm{\Delta }}logCP{M}_{m}$ indicates the difference between observed log_10_CPM and expected log_10_CPM for miRNA *m*; *f* indicates the random forest regression function; and *x*_*m*,*i*_ indicates the nucleotide of miRNA *m* at position *i*. Only the first 3 bases (*i* = 1 to 3) and the last 3 bases (*i* = −3 to −1) of each miRNA were considered in this model.

Correction of miRNA abundances was done by subtracting ${\rm{\Delta }}logCP{M}_{m}$ from the experimental log_10_CPM for each miRNA. The corrected read count was then converted into a corrected pseudo-count (non-integer) by taking a power of 10, via:

$$Corrected\,count={10}^{(lo{g}_{10}CP{M}_{m,exp}-{\rm{\Delta }}lo{g}_{10}CP{M}_{m})}$$

where ${\rm{\Delta }}logCP{M}_{m}$ indicates the correction factor derived from the model for miRNA *m* and ${\mathrm{log}}_{10}{{\rm{CPM}}}_{{\rm{m}},\exp }$ indicates the experimental log_10_CPM for miRNA *m*. Codes have been deposited in GitHub: https://github.com/wckdouglas/tgirt_smRNA.

### Comparison of TGIRT-seq of miRNAs to established small RNA-seq methods

miRNA count tables for 4N ligation, NEXTflex, TruSeq, NEBNext and CleanTag were downloaded from the National Center for Biotechnology Information (NCBI) Sequence Read Archive (SRA accession number SRP126845^36^), and counts from the 962 Miltenyi miRXplore RNAs were extracted for the comparisons. Raw reads obtained by the TGIRT-CircLigase method^6^ were downloaded from NCBI (SRA accession number SRR833775^6^) and aligned to the Miltenyi miRXplore reference sequences using Bowtie2 (settings local -D 20 -R 3 -N 0 -L 8 -i S,1,0.50 -k 5  --norc --no-mixed --no-discordant) to generate a miRNA count table. miRNA counts from TGIRT-seq datasets and the downloaded datasets were normalized to CPM for the comparisons. The predicted RNA folding and co-folding patterns and minimum free energies were computed by the ViennaRNA package^55^.

### Accession number

The datasets generated and analyzed in the current study are available in the National Center for Biotechnology Information Sequence Read Archive under SRA accession number SRP168562.

## Supplementary information

Supplementary Information
