# Supplementary material for: Improved TGIRT-seq methods for comprehensive transcriptome profiling with decreased adapter dimer formation and bias correction
Source: Sci Rep. 2019 May 28;9:7953. doi: 10.1038/s41598-019-44457-z (PMC6538698; doi:10.1038/s41598-019-44457-z)
Supplement: Supplementary file 1 — Supplementary Information [file 41598_2019_44457_MOESM1_ESM.pdf]

Supplementary Information

Improved TGIRT-seq methods for comprehensive transcriptome profiling with decreased adapter dimer formation and bias correction

Hengyi Xu,[1,2,3] Jun Yao,[1,2,3] Douglas C. Wu[1,2,3] and Alan M. Lambowitz[1,2]

[1]Institute for Cellular and Molecular Biology
University of Texas at Austin
Austin Texas, 78712, USA

[2]Department of Molecular Biosciences,
University of Texas at Austin, Austin Texas, 78712, USA

[3]These authors contributed equally to this work.
Correspondence and requests for materials should be addressed to A.M.L.
(lambowitz@austin.utexas.edu)

Supplementary Figure S1

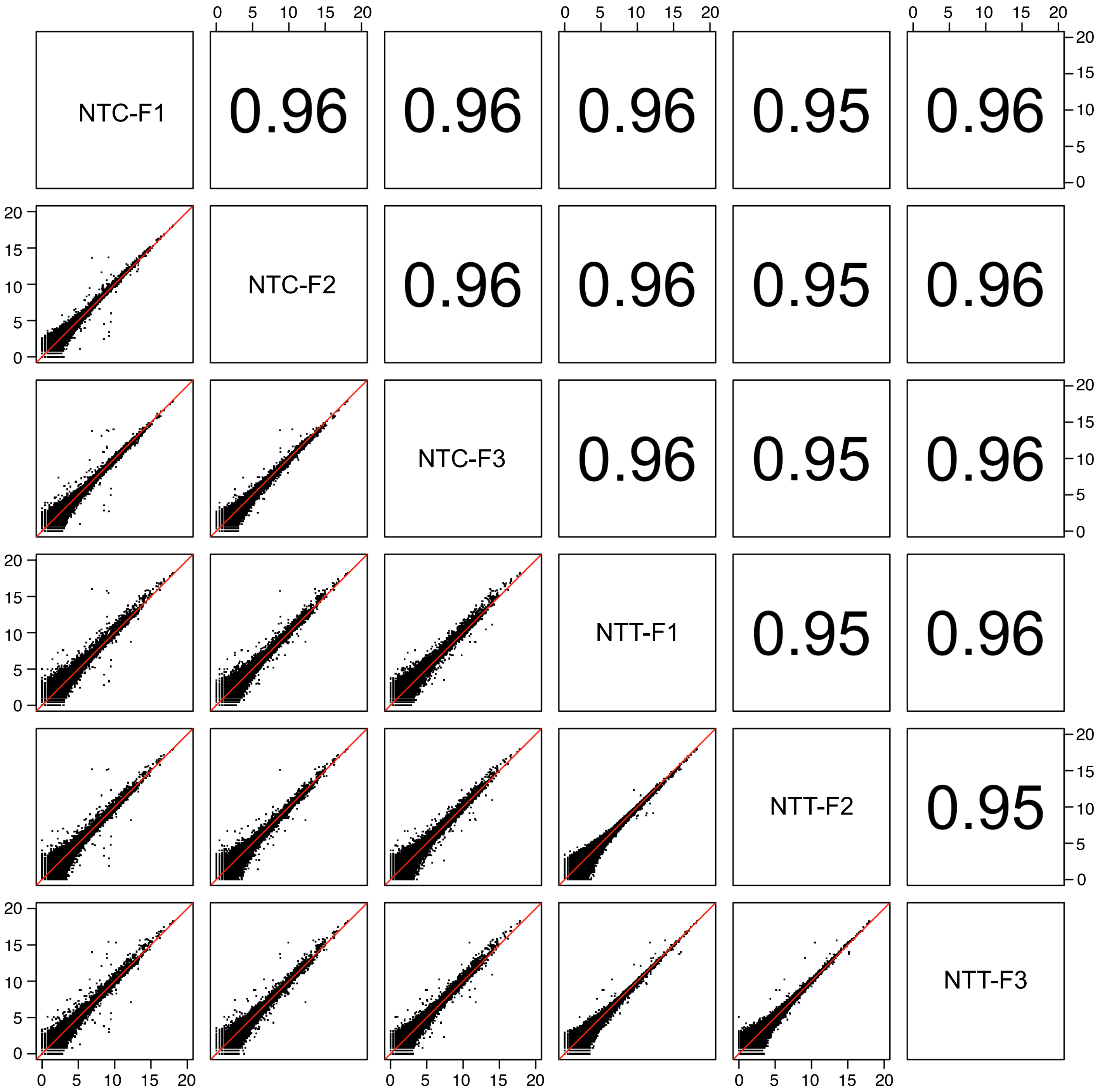

Scatter plot matrix of technical replicates in TGIRT-seq datasets of fragmented UHRR plus ERCC spike-ins using either the NTC or NTT adapter.
The x- and y-axes show DESeq2 normalized counts (log_2 scale).
Spearman's correlation coefficients (ρ) are indicated in the upper right boxes.

Supplementary Figure S2

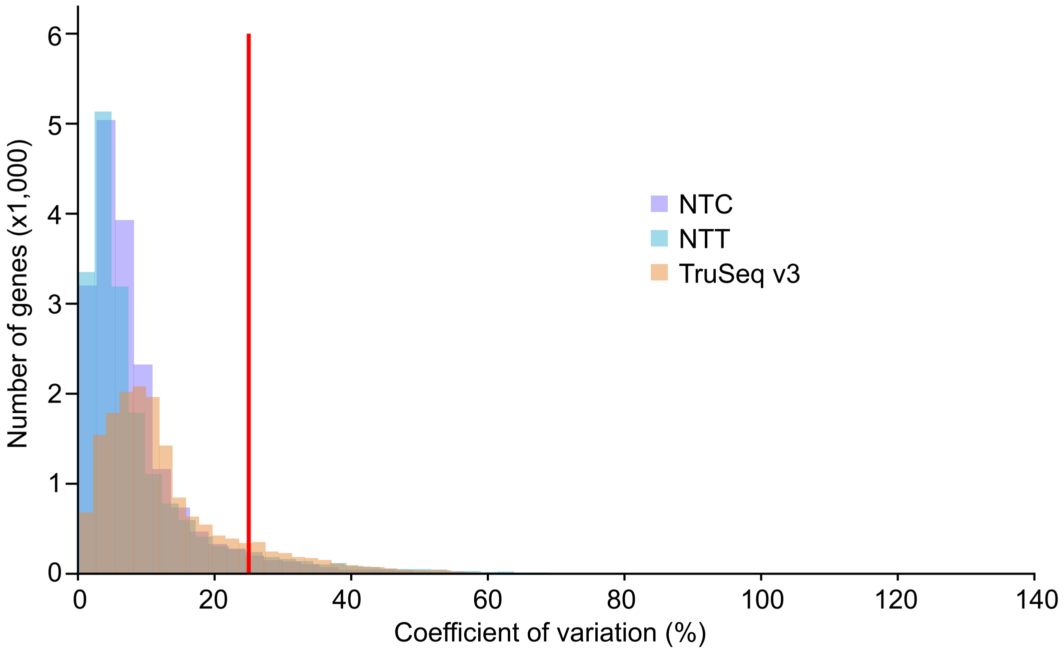

Histogram of coefficients of variation of protein-coding gene transcripts and ERCC spike-ins for TGIRT-seq of ribo-depleted fragmented UHRR samples using either the NTC or NTT adapter compared to those for TruSeq v3 (SRA accession number SRP02612630). Coefficients of variation were computed for each gene among technical replicates (n = 3 for NTC and NTT; n = 4 for TruSeq v3) and plotted as a histogram (bin size = 2.5). Only genes with DESeq2 mean normalized counts >10 were included (n = 18,457, 18,459, and 16,723 for NTC, NTT, and TruSeq v3, respectively).

Supplementary Figure S3

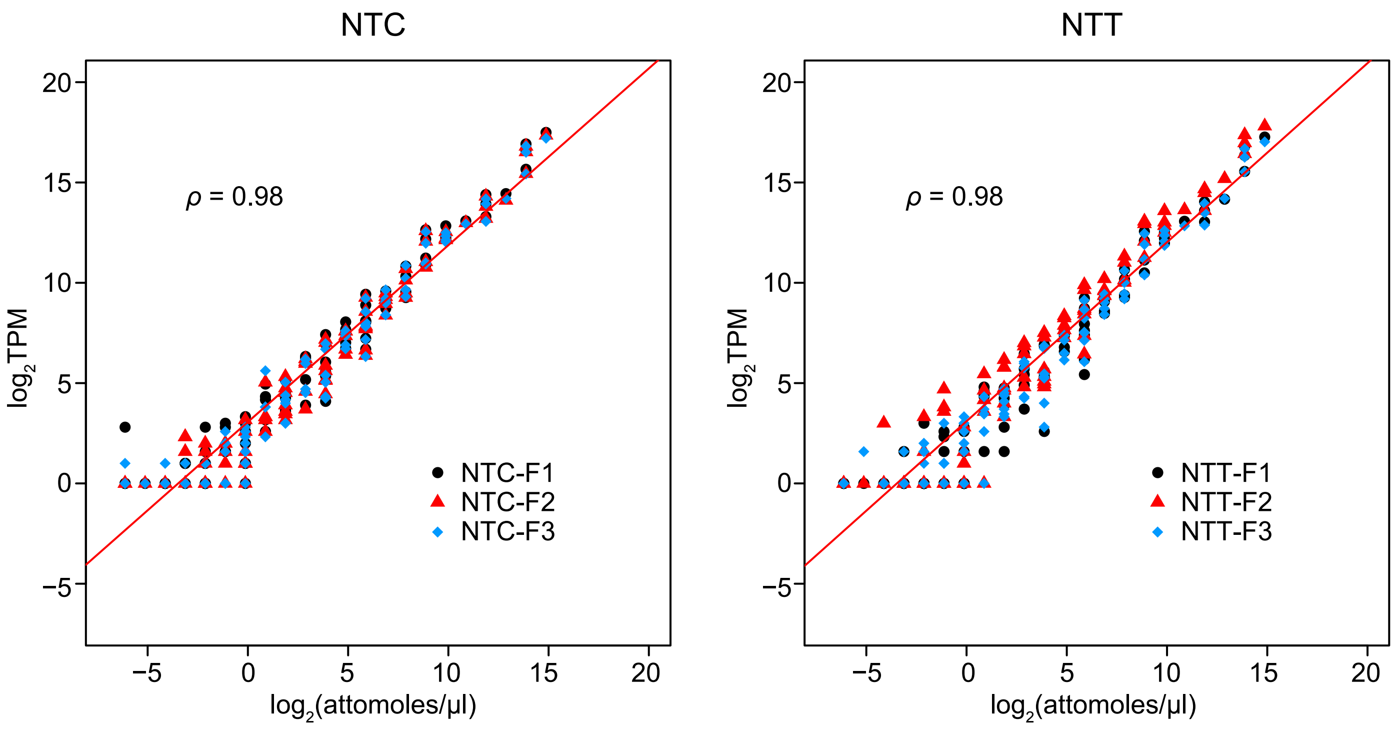

Scatter plot of ERCC spike-ins from fragmented UHRR samples using either the NTC (left) or NTT (right) adapter. The x- and y-axes show the normalized counts (TPM; $\log_2$ scale) and expected concentration (attomoles/μl; $\log_2$ scale), respectively, for each ERCC spike-in. Spike-ins from the three technical replicates are shown in different colors and symbols. Spearman's correlation coefficients (ρ) are indicated at the upper left.

Supplementary Figure S4

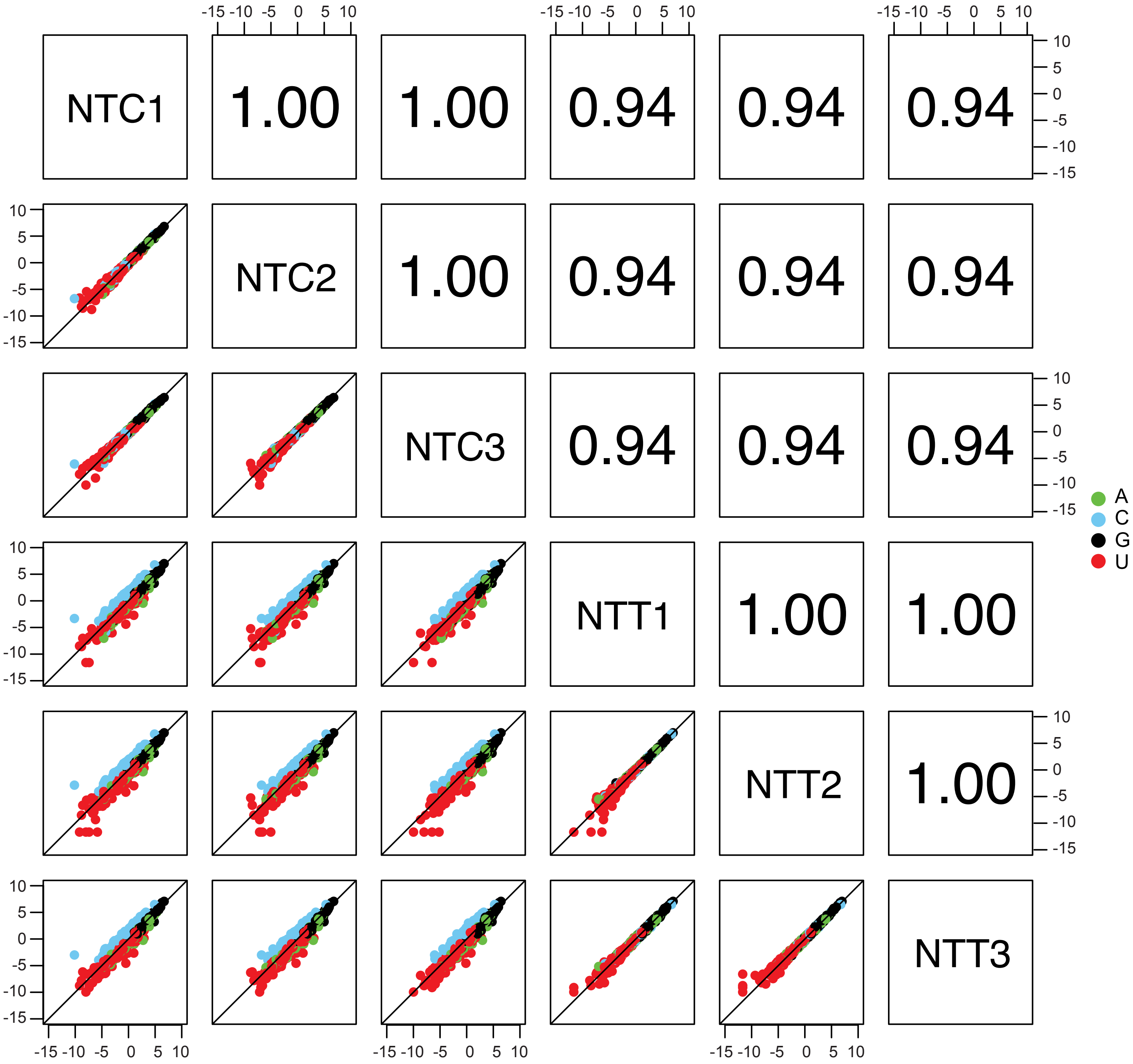

Scatter plot matrix comparing TGIRT-seq datasets obtained from the Miltenyi miRXplore miRNA reference set using either the NTC or NTT adapter (datasets NTC1-3 and NTT1-3, respectively).
The x- and y-axes show median normalized counts ($\log_2$ scale).
Spearman's correlation coefficients (ρ) are indicated in the upper right boxes.
miRNAs with different 3' nucleotides are colored coded (A, green; C, blue; G, black; U, red).

Supplementary Figure S5

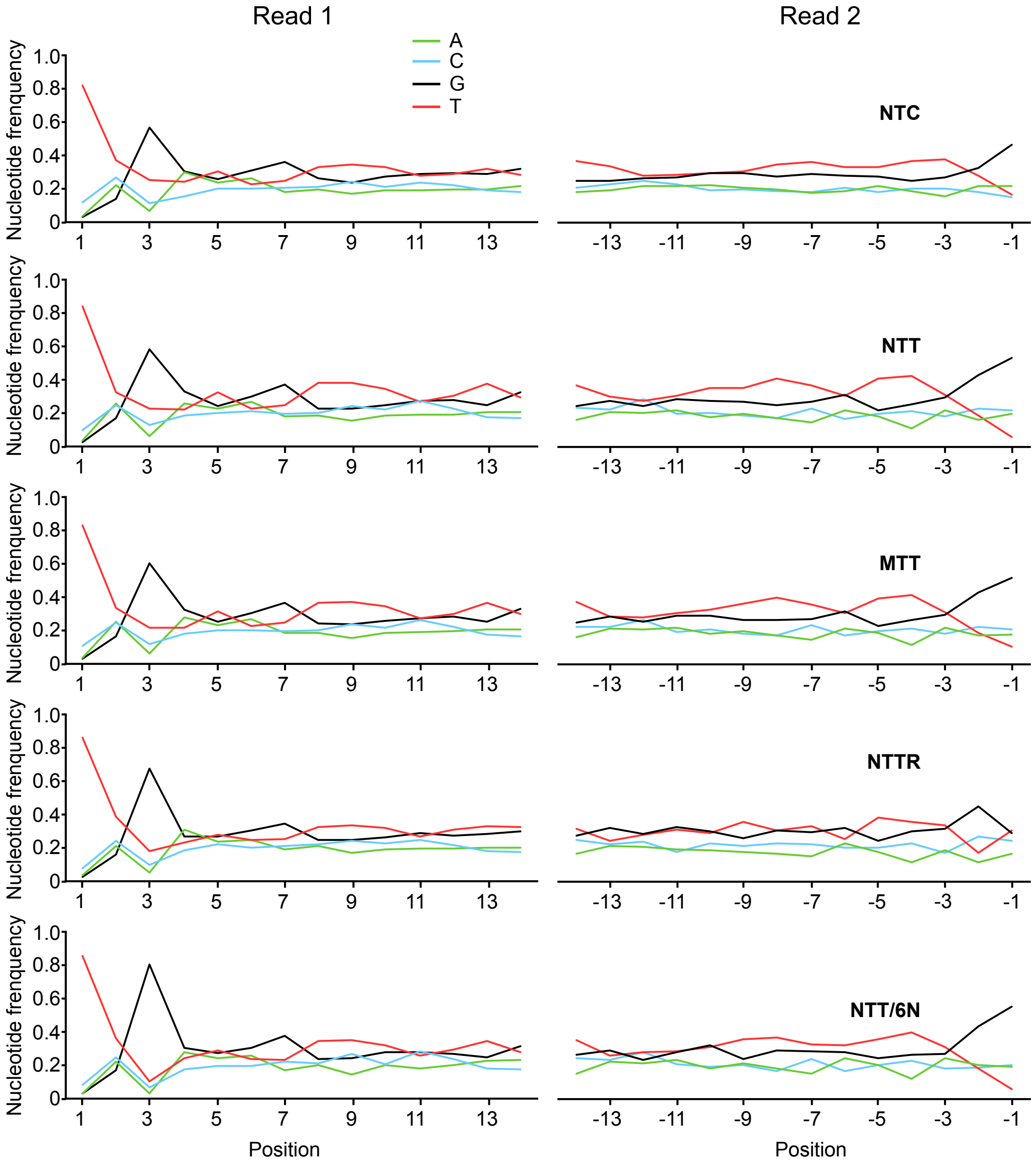

Aggregate nucleotide frequencies at the 5'-end of Read 1 (5'-RNA end; positions +1 to +14) and Read 2 (3'-RNA end; positions -1 to -14) from combined technical replicates obtained by TGIRT-seq of Miltenyi miRXplore reference set miRNAs using the NTC adapter; the NTT adapter; a modified NTT adapter mix in which the 3' A overhang is replaced with 3' diaminopurine (MTT); a modified NTT adapter mix with an altered ratio of 3' overhangs (A:C:G:T = 6.6:0.4:1:1; denoted NTTR); and the NTT adapter used together with an R1R adapter with six randomized nucleotides at its 5' end (NTT/6N).

Supplementary Figure S6

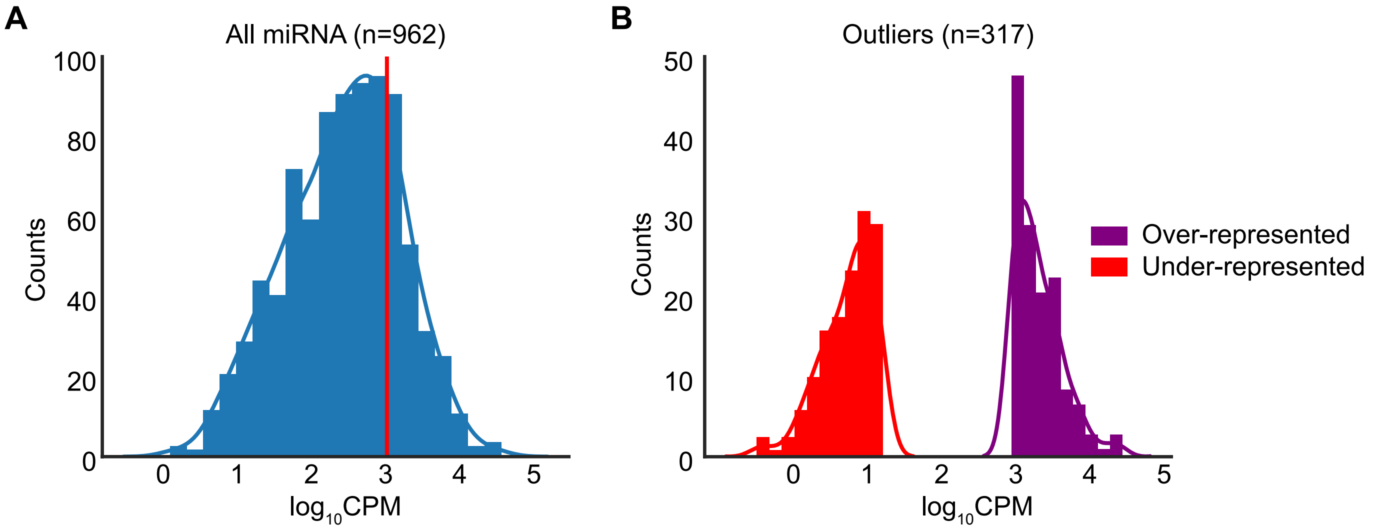

miRNAs grouped by representation of sequences in TGIRT-seq datasets obtained from the Miltenyi miRXplore miRNA reference set with the NTT adapter. (**A**) Histogram showing all 962 reference set miRNAs as a function of their $\log_{10}$CPM in combined TGIRT-seq datasets for the three technical replicates obtained using the NTT adapter. The red line indicates expected CPM value for the equimolar mix of 962 miRNAs. The histogram was computed using bin size of 0.45 and plotted on a $\log_{10}$CPM scale. (**B**) Histogram of under-represented (red) and over-represented (purple) miRNAs with $\log_{10}$CPM at least one standard deviation lower or higher than the mean $\log_{10}$CPM for all miRNAs in the reference set. The histogram was computed using bin size of 0.15 and plotted on a $\log_{10}$CPM scale.

Supplementary Figure S7

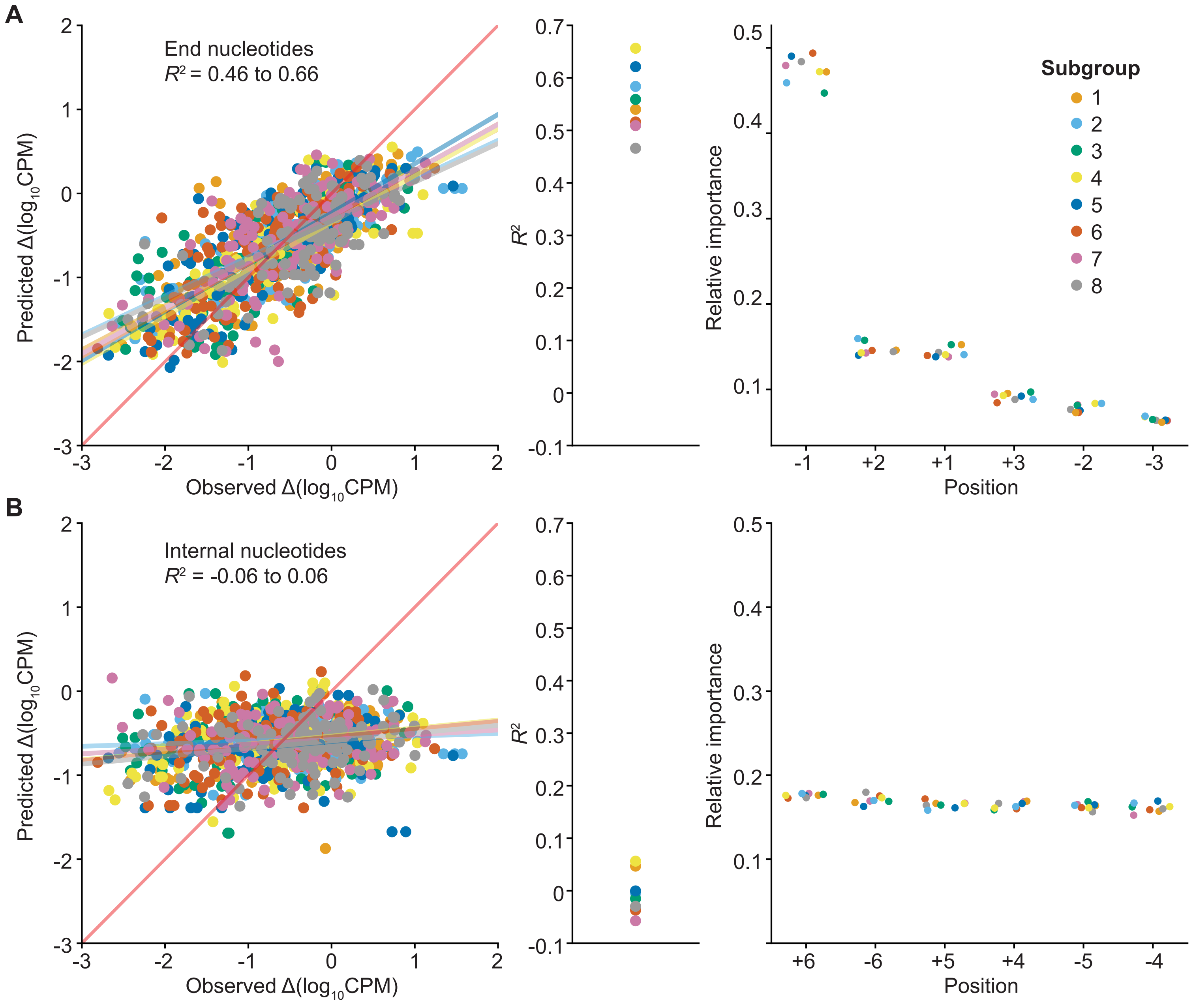

k-fold cross-validations of random forest regression bias-correction models based on the 5'- and 3'-end or internal nucleotide positions of miRNAs in combined TGIRT-seq datasets obtained with the NTT adapter for the Miltenyi miRXplore miRNA reference set. (**A**) shows results for models trained on the first 3 nucleotide positions from the 5' and 3' end of each miRNA (positions +1 to +3 and -1 to -3, respectively), and (**B**) shows results for models trained on the next 3 internal positions (positions +4 to +6 and -4 to -6, respectively). In each case, the 962 miRNAs in the dataset were randomly partitioned into 8 subgroups (120 or 121 miRNAs per group), and random forest regression models were trained against the observed measurement errors for each miRNA in a dataset comprised of 7 of the subgroups ($\Delta\log_{10}$CPM: the difference between the observed and expected $\log_{10}$CPM for that miRNA) and tested on the remaining subgroup. The plots at the left show the predicted measurement errors ($\Delta\log_{10}$CPM predicted by the random forest regression models) plotted against the observed measurement errors ($\Delta\log_{10}$CPM obtained directly from sequencing data) for each miRNA color-coded by the subgroup on which the model was tested. The fitted linear regressions for each model were plotted as similarly color-coded solid lines, with the red diagonal line indicating hypothetical perfect prediction with slope = 1 and y-intercept = 0. The plots in the middle show R^2 values for each of the models, and the plots at the right show the relative importance of each nucleotide position in each model, color-coded by the subgroup on which the model was tested.

Supplementary Figure S8

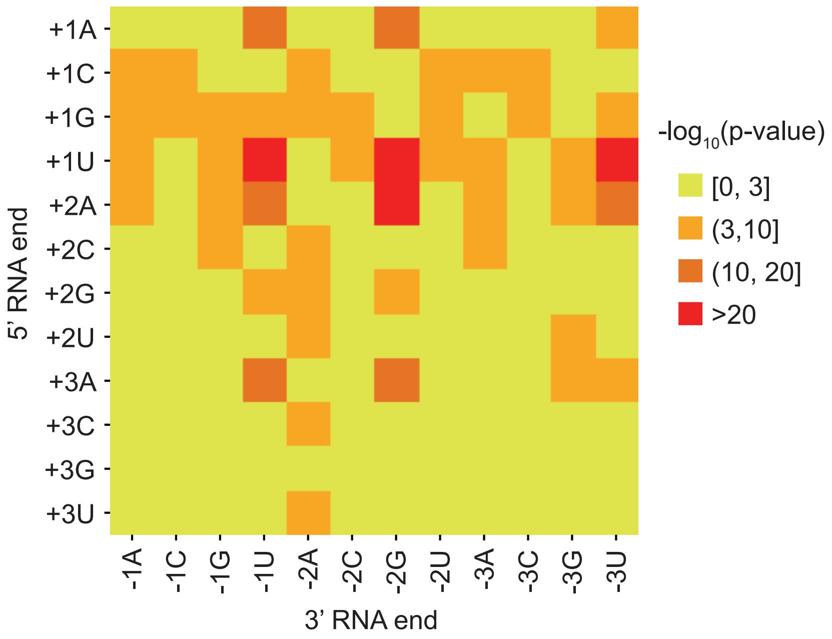

Sequence correlations between 5'- and 3'-end nucleotide positions in reference set miRNAs. Co-occurrence of nucleotide pairs from 5' and 3' ends (positions N+1 to +3 and N-1 to -3, respectively) of the miRNAs in the Miltenyi miRXplore reference set were counted, and each pair was tested against a uniform distribution (16 different nucleotide patterns per position pair) using a X^2-test. Minus $\log_{10}$p-values were adjusted for multiple comparisons by using the method of Benjamini and Hochberg[1] from the X^2-test and plotted as heat map for each of the paired nucleotide positions color-coded by significance as indicated to the right.

Supplementary Figure S9

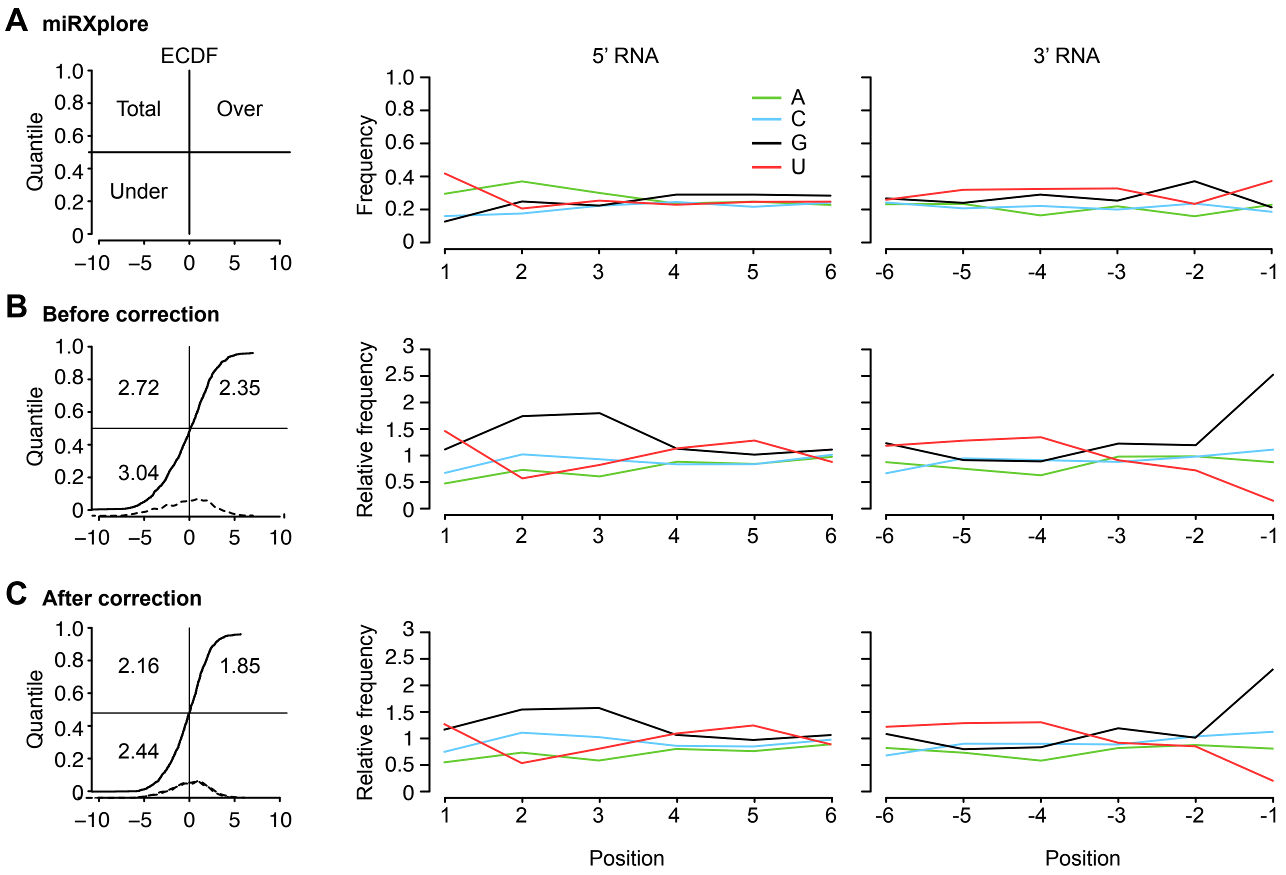

Nucleotide frequencies in a TGIRT-seq dataset of the Miltenyi miRXplore miRNA reference set obtained by using the NTT adapter before and after bias correction by reweighting of the reads. Reads were reweighted by a modification of the method of Hansen *et al.*[2] in which a reweighting algorithm was trained by using the trinucleotide frequencies at the beginning of Reads 1 and 2 of 0.5 million randomly selected read pairs mapping to ERCC spike-ins in a TGIRT-seq dataset of ribo-depleted fragmented UHRR obtained using the NTT adapter (NTT-F1). The method calculates a reweighting factor for each trinucleotide as the quotient of the frequency of that trinucleotide at unbiased internal positions (positions +7 to +37) of each read divided by the frequency of that trinucleotide at the beginning of the reads. A reweighted count for each read pair was then calculated as the geometric mean of the reweighting factors for Read 1 and Read 2. The combined datasets for the miRNA reference set (NTT1-3) before and after reweighting were used to plot both the empirical cumulative distribution function (ECDF) of the log2 median-normalized counts for each miRNA ranked from least to most abundant (left panels), and the abundance-adjusted nucleotide frequencies at the 5'-end (positions +1 to +6) and 3'-end (positions -1 to -6) for the miRNAs in the dataset relative to those in the miRNA reference set (middle and right panels). The numbers within the ECDF plots indicate the root-mean-square error (RMSE) for over-represented miRNAs (top right), under-represented miRNAs (bottom left), and all miRNAs (top left). The curve plotted as a dashed line at the bottom of the ECDF plots indicates the distribution density of the 962 miRNAs in the dataset. (A) Layout of the ECDF plot (left panel) and aggregate 5'- and 3'-nucleotide frequencies for all miRNAs in the reference set assuming equimolar concentrations of the 962 miRNAs. (B) and (C) ECDF plots (left panels) and plots of the abundance-adjusted nucleotide frequencies at the 5' and 3' ends of miRNAs in the dataset relative to those in the miRNA reference set (middle and right panels) before and after reweighting, respectively.

Supplementary Table S1. Read statistics and mapping for TGIRT-seq of the ribo-depleted, fragmented UHR RNAs.

Sample ID	NTC-F1	NTC-F2	NTC-F3	NTT-F1	NTT-F2	NTT-F3
Total reads (x10^6)	61.8	57.7	66.4	67.9	104.6	61.0
Reads (x10^6) after trimming<br>(rate)	60.0<br>(97.1%)	56.3<br>(97.6%)	65.4<br>(98.6%)	67.4<br>(99.3%)	103.9<br>(99.3%)	60.8<br>(99.8%)
Mapped reads (x10^6)<br>(rate)	53.2<br>(88.5%)	49.0<br>(87.0%)	54.9<br>(84.0%)	56.8<br>(84.2%)	89.2<br>(85.8%)	51.1<br>(84.0%)
Reads (x10^6) mapped to features<br>(rate)	49.2<br>(92.6)	45.1<br>(92.1%)	50.5<br>(91.9%)	53.0<br>(93.3%)	83.2<br>(93.3)	48.2<br>(94.3%)
ERCC total reads (x10^4)	58.8	53.6	51.7	51.1	80.3	46.8
AMPure beads clean-up (rounds)	3	3	3	1	1	1

TGIRT-seq libraries using the NTC or NTT adapter were prepared in triplicate from ribo-depleted, fragmented UHR RNAs plus ERCC spike-ins and sequenced on an Illumina NextSeq instrument to obtain the indicated numbers of 75-nt paired-end reads. The reads were trimmed to remove adapter sequences and low-quality base calls (sequencing quality cut of 20), and reads <15 nt after trimming were discarded. Trimmed reads were then mapped by using Hisat2 and Bowtie2 to a human genome reference sequence (Ensembl GRCh38) modified to include additional rRNA repeats, as described in Methods. SRA accession numbers for NTCF-1 to 3 are SRR2912443, SRR2912444 and SRR2912446[3].

Supplementary Table S2. Read statistics and mapping for TGIRT-seq of the Miltenyi miRXplore miRNA reference set.

Sample ID	NTC1	NTC2	NTC3	NTT1	NTT2	NTT3
Total reads (x10^6)	14.1	12.3	13.3	15.0	15.6	18.2
Reads (x10^6) after trimming (rate)	11.6 (82.2%)	10.1 (81.9%)	11.6 (87.6%)	12.8 (85.2%)	13.4 (85.9%)	16.1 (88.1%)
Mapped reads (x10^6) (rate)	10.1 (87.1%)	8.9 (88.1%)	10.2 (87.7%)	11.9 (93.1%)	12.4 (93.0%)	15.0 (93.4%)
Uniquely mapped reads (x10^6) (rate)	7.5 (74.1%)	6.1 (68.8%)	6.4 (62.8%)	10.4 (87.9%)	10.7 (85.9%)	13.0 (86.6%)
AMPure beads clean-up (rounds)	4	4	4	1	1	1

TGIRT-seq libraries using either the NTC or NTT adapter were prepared in triplicate from the Miltenyi miRXplore miRNA reference set and sequenced on an Illumina NextSeq instrument to obtain the indicated numbers of 75-nt paired-end reads. The reads were trimmed to remove adapter sequences and low-quality base calls (sequencing quality cut of 20), and reads <15 nt after trimming were discarded. Trimmed reads were then mapped by using Bowtie2 to a 962 miRNA reference sequences, as described in Methods.

Supplementary Table S3. Oligonucleotides used in this study

Name	Sequence and notes
**NTC R2 RNA**	5'-AGAUCGGAAGAGCACACGUCUGAACUCCAGUCAC/3SpC3/
**NTT R2 RNA**	5'-AAGAUCGGAAGAGCACACGUCUGAACUCCAGUCAC/3SpC3/
**NTC R2R DNA**	5'-GTGACTGGAGTTCAGACGTGTGCTCTTCCGATCTN-3', where N is an equimolar of A, C, G, T (obtained by hand mixing of individual oligonucleotides with A, C, G and T at their 3' end).
**NTT R2R DNA**	5'-GTGACTGGAGTTCAGACGTGTGCTCTTCCGATCTTN-3', where N is an equimolar of A, C, G, T (obtained by hand mixing of individual oligonucleotides with A, C, G and T at their 3' end).
**R1R and 6N R1R DNA**	R1R DNA: 5'-/5Phos/GATCGTCGGACTGTAGAACTCTGAACGTGT AG/3SpC3/. The R1R oligonucleotide was pre-adenylated. as described in Nottingham et al.[2]. For 6N R1R, six machine-mixed randomized nucleotides were added to the 5' end.
**Illumina multiplex PCR primer**	5'-AATGATACGGCGACCACCGAGATCTACACGTTCAGAGTTCTA CAGTCCGACGATC-3'
**Illumina index PCR primer**	5' CAAGCAGAAGACGGCATACGAGAT BARCODE* GTGACTGGA GTTCAGACGTGTGCTCTTCCGATCT-3', where BARCODE correspond to one of the 6 nucleotide Illumina TruSeq barcode sequences.
**20-nt RNA (Fig. 2)**	5'-AGGCAAGACUUUGGCAAAGC-3'
**40-nt RNA (Fig. 2)**	5'-AGGCAAGACUUUGGCAAAGCUCGCCGGGUGGAAAGCAUUC-3'

Supplementary Reference

1. Benjamini Y. & Hochberg, Y. Controlling the false discovery rate: a practical and powerful approach to multiple testing. *J. R. Statist. Soc.* B. **57,** 289-300 (1995).

2. Hansen, K. D., Brenner, S. E. & Dudoit, S. Biases in Illumina transcriptome sequencing caused by random hexamer priming. *Nucleic Acids Res.* **38**, e131; https://doi.org/10.1093/nar/gkq224 (2010).

3. Nottingham, R. M. *et al.* RNA-seq of human reference RNA samples using a thermostable group II intron reverse transcriptase. *RNA.* **22,** 597-613 (2016).
